# Scenario-based assessment of fecal pathogen sources affecting bathing water quality: novel treatment options to reduce norovirus and *Campylobacter* infection risks

**DOI:** 10.3389/fmicb.2024.1353798

**Published:** 2024-04-02

**Authors:** Annastiina Rytkönen, Päivi Meriläinen, Kristiina Valkama, Anna-Maria Hokajärvi, Josefiina Ruponen, Jarkko Nummela, Harri Mattila, Tiina Tulonen, Rauni Kivistö, Tarja Pitkänen

**Affiliations:** ^1^Department of Food Hygiene and Environmental Health, Faculty of Veterinary Medicine, University of Helsinki, Helsinki, Finland; ^2^Department of Health Security, Finnish Institute for Health and Welfare, Kuopio, Finland; ^3^Lammi Biological Station, Faculty of Biological and Environmental Sciences, University of Helsinki, Helsinki, Finland; ^4^Bio Research Unit, Häme University of Applied Sciences, Hämeenlinna, Finland

**Keywords:** quantitative microbial risk assessment, scenario-based risk assessment, bathing water quality, fecal pathogens, waterborne pathogens, water treatment, norovirus, *Campylobacter*

## Abstract

Wastewater discharge and runoff waters are significant sources of human and animal fecal microbes in surface waters. Human-derived fecal contamination of water is generally estimated to pose a greater risk to human health than animal fecal contamination, but animals may serve as reservoirs of zoonotic pathogens. In this study, quantitative microbial risk assessment (QMRA) tools were used to evaluate the hygienic impact of sewage effluents and runoff water from municipalities and animal farms on surface and bathing waters. The human-specific microbial source tracking (MST) marker HF183 was used to evaluate the dilution of fecal pathogens originating from the sewage effluent discharge to the downstream watershed. As novel risk management options, the efficiency of UV-LED disinfection and wetland treatment as well as biochar filtration was tested on-site for the contamination sources. According to the dilution pattern of the MST marker HF183, microbes from wastewater were diluted (2.3–3.7 log10) in the receiving waters. The scenario-based QMRA revealed, that the health risks posed by exposure to human-specific norovirus GII and zoonotic Campylobacter jejuni during the bathing events were evaluated. The risk for gastroenteritis was found to be elevated during wastewater contamination events, where especially norovirus GII infection risk increased (1–15 cases per day among 50 bathers) compared with the business as usual (BAU) situation (1 case per day). The noted C. jejuni infection risk was associated with animal farm contamination (1 case per day, versus 0.2–0.6 cases during BAU). Tertiary treatment of wastewater with wetland treatment and UV-LED disinfection effectively reduced the waterborne gastroenteritis risks associated with bathing. Based on the experiences from this study, a QMRA-based approach for health risk evaluations at bathing sites can be useful and is recommended for bathing site risk assessments in the future. In case of low pathogen numbers at the exposure sites, the MST marker HF183 could be used as a pathogen dilution coefficient for the watershed under evaluation. The full-scale implementation of novel tertiary treatment options at wastewater treatment plants (WWTPs) as well as on-site runoff water treatment options should be considered for infection risk management at locations where scenario-based QMRA implies elevated infection risks.

## 1 Introduction

Fecal contamination occasionally occurs at bathing sites. In the bathing season of 2018, 85% of bathing sites in Finland and in the European Union (EU) were considered to have excellent water quality (European Parliament and Council, 2006; European Environment Agency, 2019). However, three bathing water outbreaks with a total of 168 gastroenteritis cases were reported in the same year in Finland (Pihlajasaari et al., 2021). At the same time, 62% of monitored bathing sites in the USA did not receive any hygienic safety notifications indicating good bathing water quality and safe bathing environment (United States Environmental Protection Agency, 2012, 2019). Wastewater discharge and runoff waters are significant sources of fecal microbes, transmitting both human and animal pathogens to surface waters. Human-derived fecal contamination of water is generally estimated to pose a greater risk to human health than animal fecal contamination (Soller et al., 2010a,b; Wade et al., 2022). However, some animals, such as cattle and horses, may act as reservoirs for zoonotic pathogens, for example, *Campylobacter* spp. (Moriarty et al., 2015; Rapp et al., 2020; Espunyes et al., 2021; Paruch and Paruch, 2022). For reference, agricultural runoff was evaluated as the main pollution source in 36 bathing water pollution cases in the USA in 2021 (United States Environmental Protection Agency, 2022).

Quantitative microbial risk assessment (QMRA) is a risk assessment method utilizing quantitative data, such as pathogen occurrence and persistence, barrier efficacy, exposure, infectivity, individual susceptibility, and disease impact (World Health Organization, 2016a). When the health hazards are recognized, exposure and health effects are assessed, and the risks are characterized, the QMRA approach can provide detailed information on risks at the system level (World Health Organization, 2016a). QMRA is based on quantitative microbial results from the water system evaluated (World Health Organization, 2003). However, the evaluation of pathogen numbers in large water bodies is often difficult due to low pathogen numbers and dilution. Traditionally, fecal indicator bacteria, such as *Escherichia coli* and *intestinal enterococci*, have been used to evaluate water quality (World Health Organization, 2003), but they are commensals in the intestines of all warm-blooded animals, including humans, and therefore disclose nothing about the sources of fecal contamination. As the contamination source is an important factor affecting waterborne infection risks and human or animal health (Soller et al., 2010a,b; Wade et al., 2022), source identification is an important part of risk assessment (Ahmed et al., 2019).

Microbial source tracking (MST) markers are used for QMRA approaches to identify the sources of fecal contamination (Ahmed et al., 2018; Kongprajug et al., 2021). When the source of such contamination is known, exposure characterization and estimation of the potential health risks become more reliable (Ahmed et al., 2019). Norovirus, *Campylobacter jejuni*, *Campylobacter coli*, *Salmonella*, enterohemorrhagic *Escherichia coli, Cryptosporidium* spp., and *Giardia* spp. have caused outbreaks of waterborne infection in Europe and North America (Hokajärvi et al., 2013; Kulinkina et al., 2016; Kauppinen et al., 2017; Vanden Esschert et al., 2020). Norovirus and *C. jejuni* are of particular interest because they can survive for long periods in surface waters (Hokajärvi et al., 2013; Kauppinen et al., 2014). Of these pathogens, norovirus is strictly human-specific and can enter watersheds, for example, through wastewater discharge (Kauppinen et al., 2014). *Campylobacter jejuni* may originate from multiple sources, such as runoff water from cattle and poultry farms and horse facilities (Moriarty et al., 2015; Mulder et al., 2020; Rapp et al., 2020), or from wastewater discharge (Hokajärvi et al., 2013).

Wastewater treatment plants (WWTPs) are not designed for microbial removal, although the treatments can reduce the levels of intestinal microbes (Koivunen et al., 2003; Tyagi et al., 2011). However, fecal microbes can be efficiently removed when tertiary treatments, such as ultraviolet (UV) disinfection, filtration, wetland, or coagulation treatments, are applied at WWTPs (Gómez et al., 2007; Pradhan et al., 2013; Kauppinen et al., 2014; Bhatt et al., 2020). The water downstream of a WWTP discharge site might be further used for recreation, irrigation, or drinking water production, even though the effluents have a significant impact on the receiving water bodies (Czekalski et al., 2012; O’Mullan et al., 2017; Hamdhani et al., 2020). Surface waters receive effluent and runoff waters containing fecal microbes from multiple sources, which makes the microbial risks challenging to evaluate (Ahmed et al., 2019). The issue of urban pollution, such as decentralized facilities and pollution from rainwater, is also addressed in the reforming EU Urban Wastewater Treatment Directive (European Parliament and Council, 2022). Multiple factors, such as the volume and turnover rate of water, determine the actual water quality in the receiving water body in addition to the characteristics of the pollution sources (World Health Organization, 2016b).

In this study, we assessed MST marker dilution patterns from point contamination sources to the bathing sites. As multiple factors affect the hygienic status of surface waters, the use of MST marker numbers alone is not enough for estimating the waterborne infection risks (Wang et al., 2013; Boehm et al., 2015). Actually, the pathogen counts are often below the detection limits during sampling events (World Health Organization, 2017). As a novel solution, our aim was to estimate the dilution of pathogens by using the MST marker numbers to increase the accuracy of the risk assessment method. In addition, we piloted novel tertiary treatment methods to evaluate their effectiveness in reducing the infection risks, and the reduction was verified in scenario-based QMRA modeling. The objectives of this study were as follows:

1. To create a conceptual model to describe the factors affecting the bathing water quality at bathing sites and model pathogen transport from wastewater discharge areas to the bathing sites.
2. To examine the human health risks caused by fecal pathogens transported through wastewater effluents, urban runoff, and animal farm runoff to bathing sites.
3. To assess the efficiency of risk management options in addressing the human health risks at bathing sites.

## 2 Materials and methods

### 2.1 Target areas

Water samples were collected as grab samples into sterile plastic bottles from two WWTP discharge areas, three runoff water discharge areas, and two watersheds below horse farms. All the sampling sites were in the Kanta-Häme region of southern Finland.

**Figure 1:**
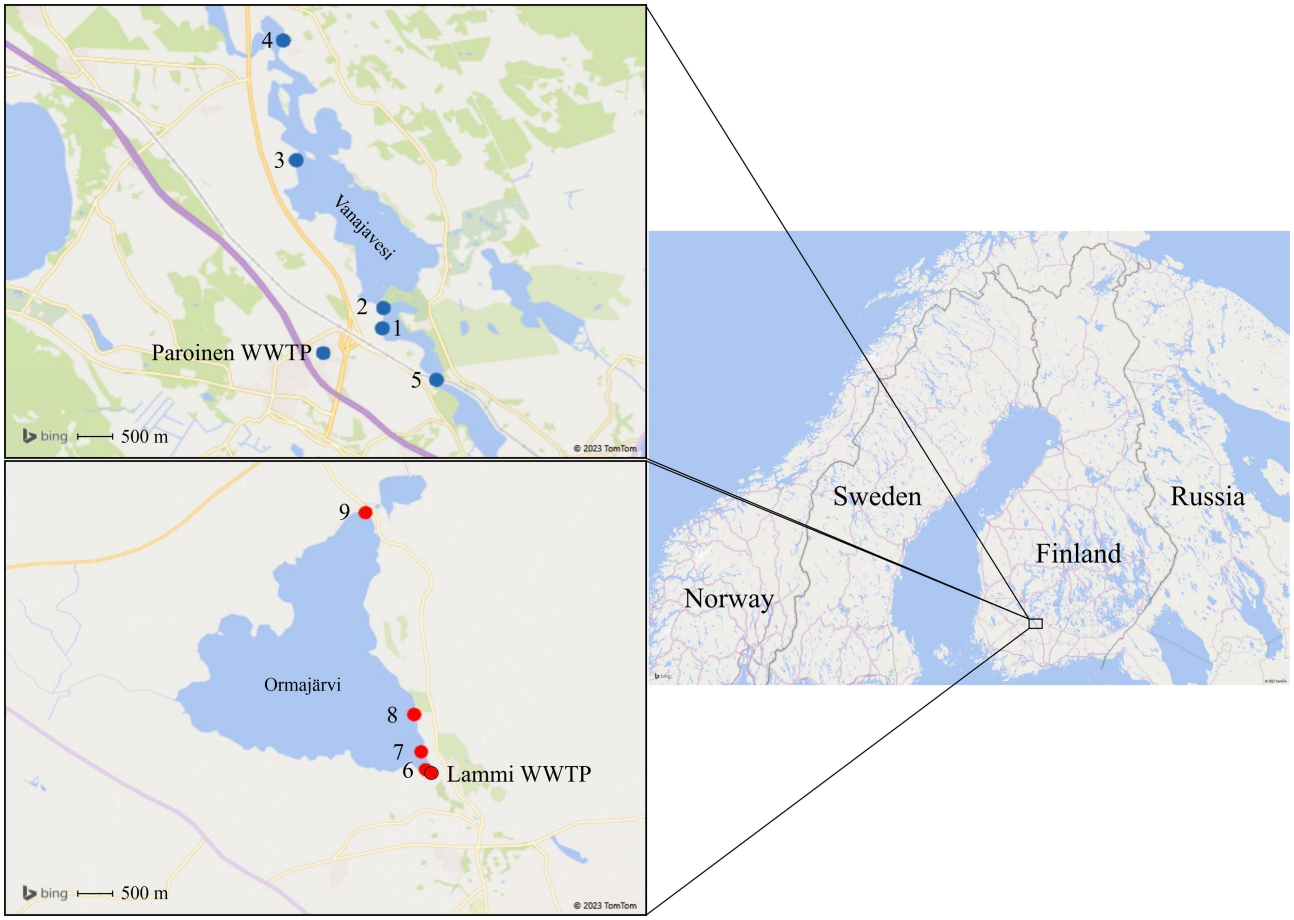
Sampling areas in Lake Vanajavesi and Lake Ormajärvi. Sampling sites 1–4 are in Lake Vanajavesi below the Paroinen WWTP. Site 1 = WWTP discharge area. Site 2 = Sampling site downstream of the discharge area. Site 3 = EU bathing area. Site 4 = Small public bathing area. Site 5 = Control site upstream of the discharge area. Sampling sites 6–9 are in Lake Ormajärvi below the Lammi WWTP. Site 6 = Wastewater outlet into a constructed wetland. Site 7 = Wastewater effluent from the constructed wetland into the lake. Site 8 = Small public bathing area. Site 9 = Small public bathing area. (Maps: 3D Map by Microsoft Excel, Microsoft Corporation 2023, United States. Microsoft product screen shots reprinted with permission from Microsoft Corporation).

The study was conducted in the areas of Lake Vanajavesi and Lake Ormajärvi in November 2019 and during the summer and autumn of 2020. The sampling sites in Lake Vanajavesi were located below the discharge area of the Paroinen WWTP, a secondary treatment plant using an activated sludge process with dissolved air flotation as a tertiary treatment (Figure 1). From sampling site 1, the water flows toward sampling site 2 (400 m downstream the discharge area) to sampling site 4 in Lake Vanajavesi. Site 3 was an EU bathing area (i.e., a bathing area included in EU-wide water quality monitoring) 3,200 m downstream the discharge area with approximately 50 visitors per day during the bathing season (June 15th to August 31st). Site 4 was a small public bathing area 5,400 m downstream the discharge area with a lower visitor frequency. Sampling site 5 was a control site 1,300 m upstream the discharge area describing the general microbial contamination in the watershed. Detailed descriptions of the sampling sites are presented in Supplementary material S1.

Sampling sites in Lake Ormajärvi were located below the discharge area of the Lammi WWTP, a secondary treatment plant using activated sludge and two-line biological and chemical coagulation (Figure 1). In addition, wetland treatment in a constructed wetland pond with a 40-day water residence time was in use (Uusheimo et al., 2018). Sampling site 8 was a small public bathing area with 500 m distance to the discharge area, with approximately 50 visitors per day during the bathing season. Sampling site 9 was a small public bathing area located on the opposite side of the lake (with 3,700 m to the discharge area), with a similar visitor frequency.

The runoff water samples were collected from one stream in suburban (sites 10 and 11) and two streams in urban (sites 12 and 13) areas in the Kanta-Häme region. Samples to investigate the microbes in animal farm runoff waters were taken from an equine college with approximately 250 horses (sites 14–16) and from a smaller riding school with approximately 50 horses (sites 17–18). Detailed descriptions of the sampling sites are presented in the Supplementary material S1.

Air temperatures (°C) during the sampling events were collected in real time from the Finnish Meteorological Institute mobile application (Finnish Meteorological Institute, 2023a). Seven-day and 30-day mean temperatures were calculated from daily mean temperatures collected from the Finnish Meteorological Institute database (Finnish Meteorological Institute, 2023b). Precipitation (mm) information on the sampling day was collected, and cumulative precipitation for days 3, 7, and 30 before the sampling was calculated (Finnish Meteorological Institute, 2023b). The weather observations were collected from the closest weather stations to Lake Vanajavesi (approximately 8 km) and Lake Ormajärvi (approximately 6 km). The weather observations are presented in Supplementary Table S1.

### 2.2 Microbial load assessment

Waterborne pathogens and fecal indicator microbes were analyzed from the collected water samples to assess the microbial load in the studied areas. The number of microbial analyses at each sampling site is presented in Supplementary Table S2. Thermotolerant *Campylobacter* spp. were determined from sample volumes ranging from 1 ml to 1,000 ml depending on the level of contamination in the samples according to ISO, 17995:2019 (International Organization for Standardization, 2019) by using Preston and Bolton broths (Oxoid, UK) and modified charcoal cefoperazone deoxycholate agar (mCCDA, Oxoid, UK). The hippurate test was used to identify *C. jejuni,* and thermotolerant *Campylobacter* species were identified with matrix-assisted laser desorption ionization–time-of-flight mass spectrometry (MALDI-TOF MS) using a MALDI Biotyper® smart device (Bruker, USA).

*Salmonella* was determined from sample volumes ranging from 1 mL to 1,000 mL depending on the level of contamination in the samples by using the standard method ISO, 19250:2010 (International Organization for Standardization, 2010). The pre-enrichment was performed by incubating the samples in buffered peptone water (BPW, Oxoid, UK). *Salmonella* enrichment was carried out with modified semi-solid Rappaport-Vassiliadis agar (MSRV, Oxoid, UK) at 41.5°C for 24 ± 3 h and recognition on xylose lysine deoxycholate (XLD, Oxoid, UK) and Brilliance Salmonella agar (BRS, Oxoid, UK) at 36 ± 2°C for 24 ± 3 h.

Indicator microbes were analyzed from sample volumes ranging from 0.1 to 100 ml depending on the level of contamination in the samples. *Escherichia coli* and coliform bacteria were determined using the Colilert®-18 Quanti-Tray method (IDEXX Laboratories, USA) according to the standard ISO, 9308-2:2012 (International Organization for Standardization, 2012). Intestinal enterococci were determined with the membrane filtration method according to the standard ISO, 7899-2:2000 (International Organization for Standardization, 2000). Sulfite-reducing clostridia were analyzed according to the standard ISO, 6461-2:1986 (International Organization for Standardization, 1986) and *Clostridium perfringens* according to the standard ISO, 14189:2013 (International Organization for Standardization, 2013). F-specific and somatic coliphages were determined as described in United States Environmental Protection Agency method 1,602 (United States Environmental Protection Agency, 2001).

Adenoviruses, noroviruses (genotypes I and II), sapoviruses, and MST markers were determined from the samples by quantitative polymerase chain reaction (qPCR). The qPCR assays were performed using the QuantStudio 6 Flex real-time PCR system (Applied Biosystems, Thermo Fisher Scientific, USA). Detailed information and the performance characteristics of the qPCR analyses are presented in Supplementary material S2 and Supplementary Tables S3, S4. For adenovirus, norovirus, and sapovirus analyses of surface water samples, a maximum of 2 L of the samples were prefiltered through 2-μm glass fiber filter (Merck KGaA, Germany). After prefiltration, the maximum volume of the filtrate of each sample was concentrated, and the nucleic acids extracted as described by Kauppinen et al. (2018). Briefly, the viruses were eluted in 50 mM glycine buffer (pH 9.5) containing 1% beef extract (MP Biomedicals, USA), and the eluate was neutralized with HCl. The eluate was concentrated with a microconcentrator (Vivaspin 2, Sartorius, Germany) to approximately 200 μL, and nucleic acids were extracted from the concentrate with High Pure Viral RNA (norovirus, sapovirus) and High Pure Viral Nucleic Acid (adenovirus) kits (Roche Molecular Biochemicals, Germany).

Adenovirus, norovirus, and sapovirus analyses from the 2-L samples were concentrated with the dead-end ultrafiltration (DEUF) method, as described by Inkinen et al. (2019). The DEUF eluate was filtered through a Millipore Express Plus (47 mm, 0.22 μm) filter (Merck KGaA, Germany), and polyethylene glycol (PEG) precipitation was used as the secondary concentration method for the filtrate. NaCl (Fisher Scientific, USA) up to a final concentration of 0.9 M and PEG 8,000 (Fisher Scientific, USA) up to a final concentration of 12% were mixed with the filtrate. The filtrates were incubated at 4°C for at least 2 h and centrifuged with swing-out rotor with 10,000 x g for 30 min at 4°C. The pellet was mixed with 0.01 M phosphate buffered saline (PBS, Fisher Scientific, USA) and stored at −80°C until nucleic acid extraction. Nucleic acids were extracted from max. 2.5 ml of pellet with a High Pure Viral Nucleic Acid Large Volume kit (Roche Molecular Biochemicals, Germany). The qPCR analysis for adenovirus (Jothikumar et al., 2005) was executed using TaqMan Environmental Master Mix 2.0 (Applied Biosystems, Thermo Fisher Scientific, United States). The presence of norovirus GI and GII (Kauppinen et al., 2014), and sapovirus (Oka et al., 2006) was analyzed using 4x TaqMan Fast Virus 1-Step Master Mix (Applied Biosystems, Thermo Fisher Scientific, United States).

The presence of human fecal marker HF183 (Haugland et al., 2010) was assayed from the samples collected from sampling sites 1–5 and 7–9. The presence of horse fecal marker HorseCytB (Schill and Mathes, 2008) was assessed from the samples collected from sites 2–5, 9, 14, 17, and 18. The HF183 marker was analyzed from DNA and reverse transcribed RNA (complementary DNA, cDNA) and the HorseCytB marker was analyzed from DNA as described previously (Pitkänen et al., 2013; Inkinen et al., 2019; Rytkönen et al., 2021). In brief, samples were filtered onto 0.4-μm polycarbonate filters (as large a volume as possible, 50–1,000 mL) (Whatman Nuclepore Track-Etched Membranes, Sigma-Aldrich, USA). The nucleic acids were extracted from the filters with a Chemagic DNA Plant Kit (Perkin Elmer, USA), and RNA aliquots were purified using a TURBO DNA-free DNase kit (Invitrogen, Thermo Fisher Scientific, USA). The purified RNA was converted into cDNA by using the SuperScript IV VILO Master Mix system for RT-PCR (Invitrogen, Thermo Fisher Scientific, USA). The qPCR analyses from DNA and cDNA were executed with TaqMan Environmental Master Mix 2.0.

### 2.3 Risk management options

The efficiency of microbial removal was tested at full scale for wetland treatment, at pilot scale for disinfecting ultraviolet light-emitting diode device (UV-LED device, Led Future Ltd., Varkaus, Finland), and at laboratory and full scale for biochar filtration. Wetland treatment was the standard tertiary treatment at the Lammi WWTP, and its efficiency was tested by sampling the wastewater effluent before (site 6) and after (site 7) it drained through the constructed wetland pond with a 40-day water residence time. Sample pairs collected from the same sites (before and after treatment) in 2018 were used in removal efficiency calculations for *E. coli* (5 pairs), enterococci (6 pairs), norovirus GII (3 pairs), and *C. jejuni* (4 pairs).

UV-LED experiments were executed at WWTP discharge sites 1 and 7 (Supplementary Table S5). The samples were taken from the sewage effluent at the sites 1 and 7. The effluents were treated with two flow rates: 300 L/h (dose 10.4 mJ/cm^3^) and 600 L/h (dose 5.2 mJ/cm^3^). Wavelength used was 275 ± 0.5 nm.

Laboratory scale biochar filtration experiments were executed by running treated wastewater collected from site 6 through differently composed biochar filter materials (Supplementary Table S5). The four materials were 50% wood-based biochar and 50% sand, 50% sludge-based biochar and 50% sand, 30% wood-based biochar and 70% sand, and 70% wood-based biochar and 30% sand. The filtration was performed with both new and reused filter materials. The filters were autoclaved and dried between the tests. The filtration time was 24 h, and the flow rate was 3 mL/min. Further, a full-scale biochar filter BioJussi (Harxo Ltd., Sastamala, Finland) or similar was in use at the horse farms. The biochar filters were installed in July 2020. The flow rates at the filters were 0.1–0.5 L/s.

### 2.4 Quantitative microbial risk assessment: bathing water contamination risks caused by sewage effluent and animal farm runoff

**Figure 2:**
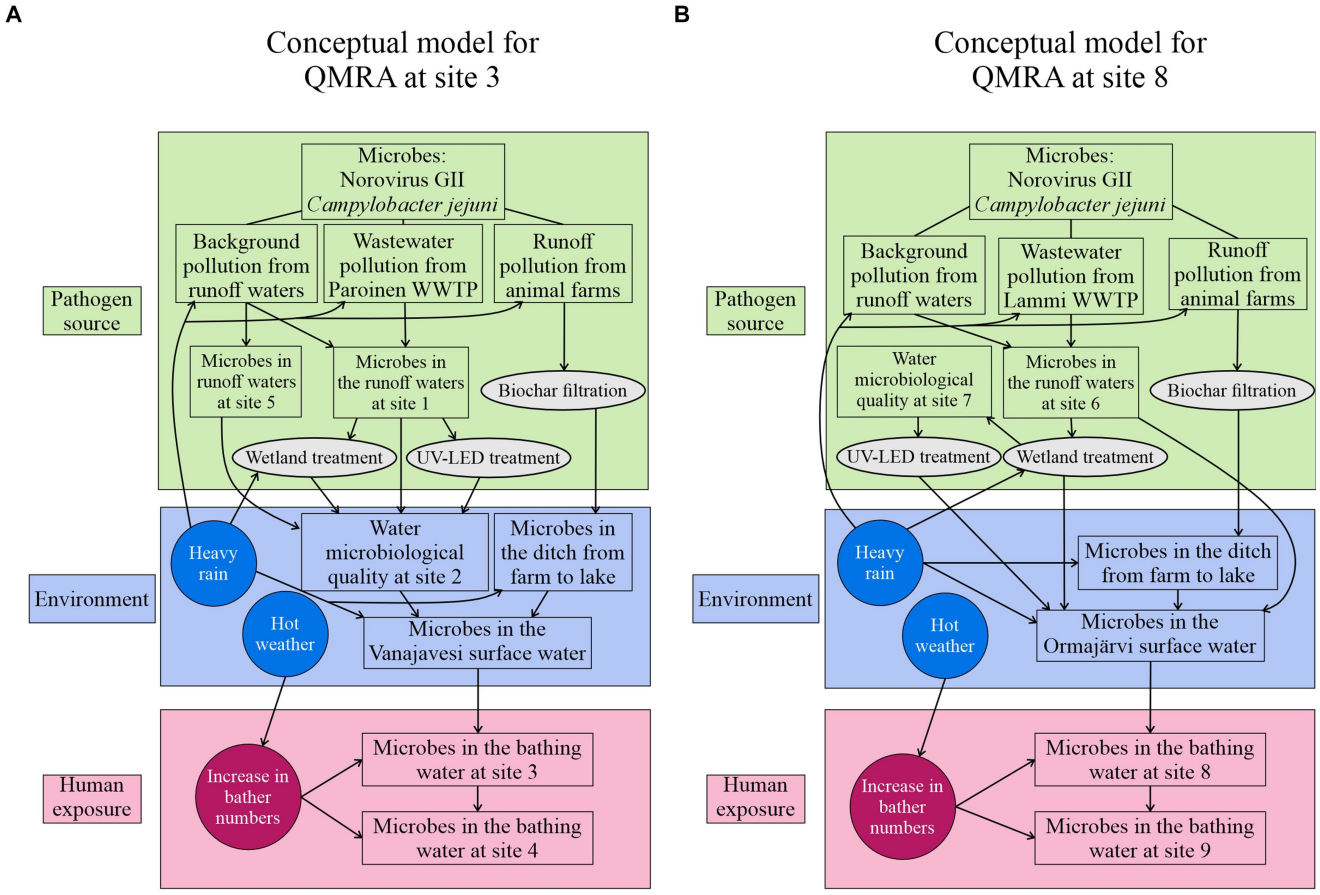
Conceptual models for the QMRA describing the norovirus and *C. jejuni* pathways to the bathing sites at **(A)** site 3 (Vanajavesi) and **(B)** site 8 (Ormajärvi). Possible events increasing the pathogen numbers are presented in the circles. Risk management options are presented in ellipses.

QMRA was performed at two bathing sites, site 3 and site 8, with approximately 50 daily visitors at both sites during the bathing season. The purpose of the QMRA was to characterize and evaluate the extent of microbial risks for bathers in a normal situation (business as usual), in potential contamination scenarios, and after novel risk management options were used. The infection risks were assessed for two waterborne pathogens, norovirus (genotype II, GII), and *Campylobacter jejuni*, which are known to occur in surface waters in Finland (Hokajärvi et al., 2013; Kauppinen et al., 2017). A conceptual model was utilized to describe the transport of pathogen and the effects of contaminant sources and risk management on the hygienic quality of the water at the bathing sites (Figure 2). The contamination and risk mitigation scenarios (Table 1) were created based on the conceptual model to evaluate the bather’s infection risks during the swimming events.

**Table 1:** Contamination and risk mitigation scenarios used in QMRA for site 3 (Lake Vanajavesi) and site 8 (Lake Ormajärvi).

Scenario	Site	Scenario description
1. Business As Usual	Site 3	Health risks the microbes can cause at site 3 in a normal situation (without wetland treatment of wastewater). Executed according to the microbe numbers determined from site 3. No known contamination or fault situations happened at the WWTP during the sampling.
	Site 8	Health risks the microbes can cause at site 8 in a normal situation (with wetland treatment of wastewater). Executed according to the microbe numbers determined from site 8. No known contamination or fault situations happened at the WWTP during the sampling.
2. Heatwave	Site 3	Health risks the microbes can cause on the bathing sites when the visitor number increases by 300%. Executed by increasing the daily visitor number at the bathing sites from 50 to 200 bathers per day.
	Site 8	
3a. Wastewater contamination with wetland treatment	Site 3	Health risks the microbes can cause at site 3, when wastewater pollution from the Paroinen WWTP increases by 50% and wetland treatment is in use. Executed by increasing the number of microbes determined from site 1 by 50% and subtracting the dilution calculated according to the HF183 marker numbers and the reduction caused by wetland treatment. The calculated number of microbes was added to the number of microbes determined from site 3 in Scenario 1.
	Site 8	Health risks the microbes can cause at site 8, when wastewater pollution from the Lammi WWTP increases by 50%. Executed by increasing the number of microbes determined from site 7 (effluent after wetland treatment) by 50% and subtracting the dilution calculated according to the HF183 marker numbers. The calculated number of microbes was added to the number of microbes determined from site 8 in Scenario 1.
3b. Wastewater contamination without wetland treatment	Site 3	Same than scenario 3a except without the reduction caused by wetland treatment.
	Site 8	Same than scenario 3a except executed by increasing the number of microbes determined from site 6 (effluent before wetland treatment) by 50%.
3c. Wastewater contamination and UV-LED disinfection	Site 3	Same than scenario 3b except with the reduction caused by UV-LED disinfection.
	Site 8	Same than scenario 3a except with the reduction caused by UV-LED disinfection
4. Urban runoff	Site 3	Health risks the microbes from an imaginary suburban area can cause at site 3. Executed by adding 5% of the number of microbes determined from runoff sites 10–13 to the number of microbes determined from site 3 in Scenario 1.
	Site 8	Health risks the microbes from an imaginary suburban area can cause at site 8. Executed by adding 5% of the number of microbes determined from runoff sites 10–13 to the number of microbes determined from site 8 in Scenario 1.
5a. Animal farm runoff	Site 3	Health risks the microbes from an imaginary animal farm can cause at site 3. Executed by adding 5% of the number of microbes determined from site 14 ditch to the number of microbes determined from site 3 in Scenario 1.
	Site 8	Health risks the microbes from an imaginary animal farm can cause at site 8. Executed by adding 5% of the number of microbes determined from site 14 ditch to the number of microbes determined from site 8 in Scenario 1.
5b. Animal farm runoff and biochar filtration	Site 3	Same than scenario 5a except with the reduction caused by the biochar filtration.
	Site 8	Same than scenario 5a except with the reduction caused by the biochar filtration.

### 2.5 Exposure assessment

The microbial numbers at the sampling sites were determined as explained in chapter 2.2. Microbial numbers were presented per 1,000 mL in the risk assessment calculations. The results below the limit of detection (LOD) or below the limit of quantitation (LOQ) were determined with equations Eq. (1) and Eq. (2), respectively (Supplementary material S3). For *C. jejuni,* semi-quantitative result estimates were used. The health effect used in the risk assessment was GI infection, and the exposure mechanism considered was swallowing the water while bathing.

The decrease in pathogen numbers was modeled by using the mean DNA and RNA GC numbers for the MST marker HF183. Both DNA and RNA aliquots were used to increase analytical sensitivity (Pitkänen et al., 2013; Rytkönen et al., 2021) and to further confirm the dilution pattern of the marker in the watershed. The decrease was calculated for the Lake Vanajavesi area by subtracting the mean log_10_ HF183 GC number at site 3 from the mean log_10_ HF183 GC number at site 1. For Lake Ormajärvi area, the decrease was calculated by subtracting the mean log_10_ HF183 GC number at site 8 from the mean log_10_ HF183 GC number at site 7.

The increase in norovirus and *C. jejuni* numbers at sites 3 and 8 during the wastewater contamination scenarios was calculated with Eq. (3) (Supplementary material S3). For the urban runoff scenario, ratios between norovirus GII and somatic coliphages and between *C. jejuni* and *E. coli* were calculated from the minimum, mean, and maximum values of these microbes measured from sites 1–3 and 6–8.

The decrease in microbial contamination during the scenarios, in which microbe removal techniques were in use, was calculated by subtracting the minimum, mean, and maximum log_10_ microbe number after the treatment from the minimum, mean, and maximum log_10_ microbe number before the treatment. For the wetland treatment scenarios, the decrease was calculated separately for norovirus GII and *C. jejuni*. For the UV-LED disinfection scenarios, the decrease was calculated separately for *E. coli*, intestinal enterococci, *C. perfringens,* and somatic coliphages. For the biochar filtration scenarios, the decrease was calculated separately for *E. coli*, intestinal enterococci, and somatic coliphages. Minimum, mean, and maximum values for the decrease in the number of microbes during different sampling times or each parallel sample were calculated. These values were used to determine the norovirus GII or *C. jejuni* numbers in the contamination scenarios. As norovirus GII and *C. jejuni* were not measured from the UV-LED disinfection and biochar filtration test samples, somatic coliphage reduction was used to describe the norovirus reduction, and *E. coli* reduction was used to describe the *C. jejuni* reduction in these scenarios.

### 2.6 QMRA tool: bathing water guide

The health risk assessment for sites 3 and 8 was performed using the Bathing Water Guide online tool (Opasnet, 2019). The Bathing Water Guide tool can be used to evaluate the microbial risks in natural bathing waters and includes six reference pathogens, *C. jejuni*, *E. coli* O157:H7, rotavirus, norovirus, *Cryptosporidium parvum,* and *Giardia lamblia*. The Bathing Water Guide performs the health risk assessment based on inputs of the daily number of bathers and the number of pathogens in the water and calculates the exposure, including the number of ingested microbes in different age groups, total infections for each pathogen per day, and the percentage of infected users among the overall users of the bathing site. The parameters used in the Bathing Water Guide tool are presented in the Supplementary Table S6. The health risk assessment for sites 3 and 8 was carried out with the user numbers at each bathing site and the maximum, minimum, and mean norovirus GII and *C. jejuni* numbers estimated for each scenario.

### 2.7 Statistical tests

All microbial data above the LOD were logarithmically transformed (log_10_) before further statistical analysis, as the original data did not follow a normal distribution. Microbe numbers below the LOD were handled as not detected and included in the statistical analyses. The impact of weather (temperature and rainfall) on the microbe numbers in the surface waters was analyzed with the Spearman rank correlation test for *C. jejuni*, norovirus, *E. coli*, intestinal enterococci, and somatic coliphages. The significance of differences between the number of microbes detected before and after wetland treatment, UV-LED disinfection, and biochar filtration was evaluated with the Kruskal–Wallis test. The difference was considered statistically significant when *p* < 0.05. All the statistical tests were conducted in SPSS Statistics (IBM Corporation 2023, United States), and figures were drawn in Microsoft Excel (Microsoft Corporation 2023, United States) and CorelDRAW (Corel Corporation 2023, United States).

## 3 Results

### 3.1 Microbial load assessment

**Figure 3:**
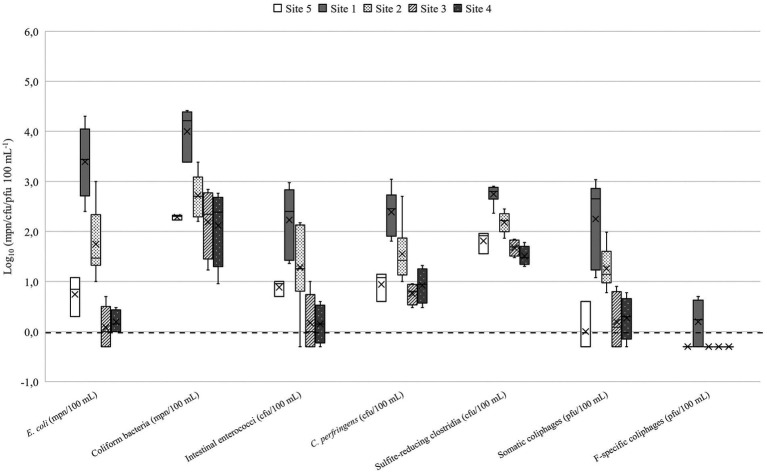
Fecal indicator microbes detected in Lake Vanajavesi. Site 1 = WWTP discharge area. Site 2 = Sampling site downstream the of discharge area. Site 3 = EU bathing area. Site 4 = Small public bathing area. Site 5 = Control site upstream the discharge area. *N* = 3–6. The mean is presented in the boxplots with a vertical line and the median with a cross. The limit of detection (LOD) log_10_ 0.0 or 1 mpn/cfu/pfu 100^−1^ is marked as a dotted line. Samples where target microbes were not detected (the results were below the LOD) are presented as 0.5 × LOD = log_10_–0.3 or 0.5 mpn/cfu/pfu 100^−1^.

**Figure 4:**
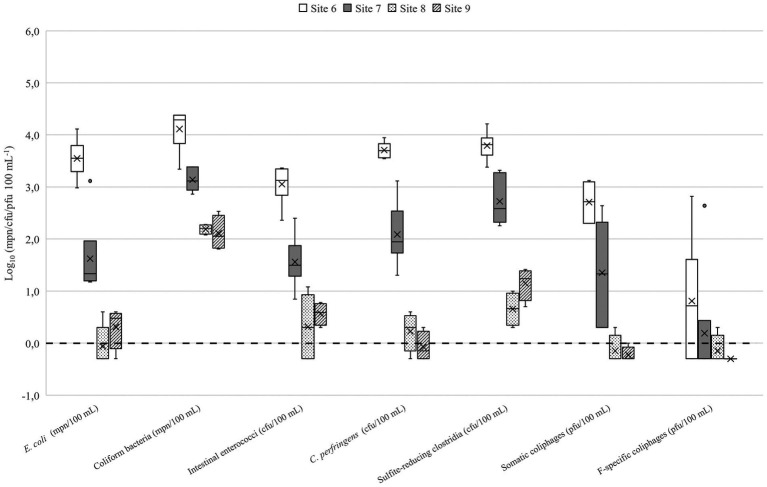
Fecal indicator microbes detected in Lake Ormajärvi. Site 6 = Wastewater outlet into the constructed wetland. Site 7 = Wastewater effluent from the constructed wetland into the lake. Site 8 = Small public bathing area. Site 9 = Small public bathing area. *N* = 3–6. The mean is presented in the boxplots with a vertical line and the median with a cross. The limit of detection (LOD) log_10_ 0.0 or 1 mpn/cfu/pfu 100^−1^ is marked as a dotted line. Samples where target microbes were not detected (the results were below the LOD) are presented as 0.5 × LOD = log_10_–0.3 or 0.5 mpn/cfu/pfu 100^−1^.

The number of microbes used in QMRA scenarios are presented in the Table 2. In lakes Vanajavesi and Ormajärvi, the fecal indicator microbe numbers were highest in the WWTP discharge areas (sites 1 and 6) (Figures 3, 4), and the number of indicator microbe numbers decreased downstream from the discharge areas. Similarly, the highest *C. jejuni*, *Salmonella*, and pathogenic virus numbers were detected in the WWTP discharge areas (Supplementary Table S7). *C. lari* and *Salmonella* were detected once at site 3. *C. jejuni*, *C. lari*, and adenovirus were detected at site 8. In the streams collecting runoff waters, the highest number of fecal indicator microbes was measured from a stream collecting urban runoff (site 13) (Supplementary Figure S1). At the horse farms, the highest numbers were detected from a well collecting runoff water from the riding school (site 17) (Supplementary Figure S2). *C. jejuni* and *Salmonella* were detected once from the equine college ditch (site 14) (Supplementary Table S7). The impact of weather on the number of microbes was determined for *C. jejuni*, *E. coli*, intestinal enterococci, and somatic coliphages (Supplementary Table S8). The number of microbes measured from the surface water samples was higher at cooler air temperatures. The strongest associations with the number of microbes were observed with the 7-day mean temperatures [*r_s_* = (−0.877) – (−0.529); *p* ≤ 0.001–0.002]. The 7-day cumulative precipitation prior to sampling was associated with the presence of *C. jejuni* (*r_s_* = 0.799; *p* = 0.002) and counts of somatic coliphages (*r_s_* = 0.495; *p* = 0.005), and *E. coli* (*r_s_* = 0.445; *p* = 0.010). The 30-day cumulative precipitation prior the sampling had a relation to increased intestinal enterococci counts (*r_s_* = 0.491; *p* = 0.004).

**Table 2:** Evaluated minimum, mean, and maximum numbers of norovirus GII (GC/1,000 ml) and *C. jejuni* (cfu/1000 ml) in different contamination scenarios at Lake Vanajavesi and Lake Ormajärvi in QMRA conducted with the Bathing Water Guide online tool (Opasnet, 2019).

Microbe	Scenario	Lake Vanajavesi				Lake Ormajärvi			
		GC/1,000 ml or cfu/1,000 ml			*N*	GC/1,000 ml or cfu/1,000 ml			*N*
		Min	Mean	Max		Min	Mean	Max	
Norovirus GII	1. Business as usual*	1.72	2.15	2.65	4	1.59	1.59	1.59	4
	2. Heatwave*	1.72	2.15	2.65	4	1.59	1.59	1.59	4
	3a. Wastewater contamination with wetland treatment	1.73	2.17	2.66	7	117.00	117.00	117.00	7
	3b. Wastewater contamination without wetland treatment	1.90	2.34	2.83	4	243.00	243.00	243.00	10
	3c. Wastewater contamination and UV-LED disinfection	1.72	2.15	2.65	6	4.05	4.05	4.05	6
	3d. Wetland treatment not in use	–	–	–	–	163.00	163.00	163.00	10
	4. Urban runoff	1.72	5.14	27.90	3	1.59	4.58	26.90	3
*C. jejuni*	1. Business as usual*	0.23	0.23	0.23	4	0.23	1.42	5.00	4
	2. Heatwave*	0.23	0.23	0.23	4	0.23	1.42	5.00	4
	3a. Wastewater contamination with wetland treatment	0.24	0.24	0.24	10	0.37	1.56	5.14	10
	3b. Wastewater contamination without wetland treatment	0.28	0.28	0.28	3	4.40	5.60	9.18	11
	3c. Wastewater contamination and UV-LED disinfection	0.23	0.23	0.23	6	0.23	1.42	5.00	6
	3d. Wetland treatment not in use	–	–	–	–	3.01	4.20	7.78	11
	4. Urban runoff	0.25	0.38	1.52	12	0.24	1.57	6.29	12
	5a. Animal farm runoff	0.24	4.50	25.20	6	0.24	5.69	30.00	6
	5b. Animal farm runoff and biochar filtration	0.23	1.23	6.11	3	0.23	1.22	6.02	6

*N*, number of observations. *Norovirus GII and *C. jejuni* numbers measured from sites 3 (Lake Vanajavesi) and 8 (Lake Ormajärvi). – Scenario not applied at the Vanajavesi sampling area.

**Figure 5:**
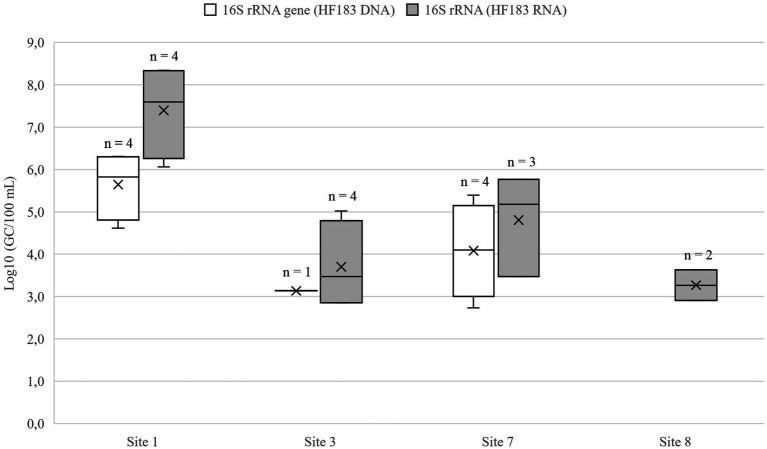
The gene copy numbers of human fecal marker HF183 in the WWTP discharge areas (sites 1 and 7) and bathing sites (sites 3 and 8) of Lakes Vanajavesi and Ormajärvi. The mean is presented in the boxplots with a vertical line and the median with a cross. *n*, Number of the samples above the LOD. No HF183 DNA was detected at the site 8.

Wastewater dilution in lakes Vanajavesi and Ormajärvi was modeled using the arithmetic mean DNA and RNA values for the HF183 marker (Figure 5). The HF183 GC numbers decreased in lake Vanajavesi from the WWTP discharge area (Site 1; 5.6–7.5 log_10_ GC/100 ml) to the bathing site (site 3; 1.9–3.7 log_10_ GC/100 ml), and in lake Ormajärvi from the WWTP discharge area (site 7; 4.1–4.8 log_10_ GC/100 ml) to the bathing site (site 8; 1.3–2.5 log_10_ GC/100 ml). The model was created by subtracting the mean GC number detected at the bathing site from the mean GC number detected at the discharge site. The arithmetic mean values of the subtractions were calculated for DNA and RNA, and these mean values were used for the wastewater contamination scenarios (Table 3). The horse-specific HorseCytB marker was detected once from the riding school runoff drain (site 17) (4.0 log_10_ GC/100 ml).

**Table 3:** HF183 gene copy (GC) numbers detected from the sampling sites presented as arithmetic mean values with the standard deviations, together with the log_10_ of mean values describing the reduction and dilution of fecal microbes from site 1 to site 3, and from site 7 to site 8.

	Log_10_ 16S rRNA gene (GC/100 ml)	Log_10_ 16S rRNA (GC/100 ml)	Log_10_ Mean (GC/100 ml)
Paroinen WWTP discharge area, site 1	5.6 ± 0.8	7.4 ± 1.1	
Bathing site, site 3	1.9 ± 0.8	3.7 ± 1.1	
Dilution (site 1 – site 3)*	3.7	3.7	3.7
Lammi WWTP discharge area, site 7	4.1 ± 1.1	4.3 ± 1.4	
Bathing site, site 8	1.3 ± 0.3	2.5 ± 1.0	
Dilution (site 7 – site 8)*	2.8	1.8	2.3

*Dilution of the marker is presented as the subtraction of the mean values.

### 3.2 Efficiency of novel risk management options

The reduction in the number of microbes was determined for wetland treatment (norovirus GII and *C. jejuni*) based on the results obtained from sampling sites 6 and 7 (Table 4). Fecal microbe numbers before and after wetland treatment are presented in the Supplementary Table S9. The pilot studies on UV-LED disinfection and biochar filtration were used to determine the reductions of *E. coli*, intestinal enterococci, somatic coliphages, and *C. perfringens* (not analyzed from the laboratory scale biochar filtration experiment samples) (Table 5). Fecal microbe numbers before and after UV-LED disinfection and biochar filtrations are presented in the Supplementary Table S10. Generally, bigger difference in microbial counts was detected from the wastewater effluent before and after the UV-LED disinfection with the flow rate of 300 L/h (10.4 mJ/cm^3^ dose) (*p* = 0.004–0.200) than with the flow rate of 600 L/h (5.2 mJ/cm^3^ dose) (*p* = 0.004–0.522). The reduction rates obtained with the 300 L/h flow rate were used in the QMRA calculations. Of the biochar filtration experiments, the biggest difference in the numbers of fecal indicator bacteria was detected from wastewater effluent before and after the sludge-based biochar filter (*p* = 0.050), but this filter material was also noticed to release phosphorus into the treated water (Supplementary Table S11). As this filter material therefore cannot be used in real life, the reduction rates of the 50% wood-based biochar filter were used for *C. jejuni* infection risk calculations, even though the number of microbes obtained after were not statistically different from the numbers before the filter (*p* = 0.127–0.275) (Table 5). The reductions obtained with the filters used in the equine college were greater with the biochar filter, but the difference between the microbial numbers before and after the filter were generally small or non-existent (*p* = 0.127–1.000) (Table 5).

**Table 4:** The log_10_ removal of fecal microbes in wetland treatment determined by using wastewater samples collected at Lammi WWTP.

Microbe	Min. (log_10_)	Mean (log_10_)	Max. (log_10_)	SD (log_10_)	*n*	*p*-value*
*E. coli*	0.37	2.28	4.86	1.24	11**	<0.001
Intestinal enterococci	0.47	1.44	2.15	0.51	12**	<0.001
Sulfite-reducing clostridia	0.37	1.07	1.96	0.56	6	0.004
*C. perfringens*	0.66	1.62	2.49	0.58	6	0.004
Somatic coliphages	0.49	1.36	2.45	0.76	6	0.010
F-specific coliphages	0.00	0.62	1.51	0.60	6	0.153
Norovirus GII	−0.04	1.05	2.95	1.13	8**	0.185
*C. jejuni*	−0.01	0.54	2.05	0.82	10**	0.385

*p*-value < 0.05 indicates significantly lower microbial counts in wastewater effluent samples collected after the wetland treatment in comparison with the samples collected before the treatment. n, Number of before and after treatment pairs analyzed. Results below LOD and LOQ determined with equations Eq. (1) and Eq. (2), respectively (Supplementary material S3). *Determined by using the Kruskal–Wallis test from microbe numbers before and after treatment (Supplementary Table S9). **Sample pairs collected from the Lammi WWTP (before and after wetland treatment) in 2018 were used in removal efficiency calculations for *E. coli* (5 pairs), enterococci (6 pairs), norovirus GII (3 pairs), and *C. jejuni* (4 pairs).

**Table 5:** The log_10_ removal of fecal indicator microbes determined in **(A)** UV-LED disinfection experiment, **(B)** laboratory scale biochar filtration experiment, and **(C)** full-scale biochar filtration at the equine college.

(A) UV-LED disinfection																				
Tertiary treatment	*E. coli* (log_10_)					Intestinal enterococci (log_10_)					*C. perfringens* (log_10_)					Somatic coliphages (log_10_)				
	Min.	Mean	Max.	SD	*p*	Min.	Mean	Max.	SD	*p*	Min.	Mean	Max.	SD	*p*	Min.	Mean	Max.	SD	*p*
UV-LED 300 L/h (*N* = 6*)	1.50	1.83	2.32	0.30	0.016	1.07	1.29	1.57	0.20	0.006	0.09	0.19	0.06	0.06	0.200	1.44	1.67	2.08	0.21	0.004
UV-LED 600 L/h (*N* = 6)	0.47	0.77	1.03	0.25	0.150	0.52	0.65	0.86	0.11	0.006	−0.05	0.06	0.16	0.08	0.522	1.06	1.56	2.08	0.44	0.004

(B) Laboratory scale biochar filtration															
Tertiary treatment	*E. coli* (log_10_)					Intestinal enterococci (log_10_)					Somatic coliphages (log_10_)				
	Min.	Mean	Max.	SD	*p*	Min.	Mean	Max.	SD	*p*	Min.	Mean	Max.	SD	*p*
50% wood-based biochar (*N* = 3)	0.57	0.64	0.75	0.08	0.127	0.41	0.51	0.62	0.09	0.275	NA	NA	NA	NA	NA
50% sludge-based biochar (*N* = 3)	0.57	0.85	1.08	0.21	0.050	0.53	0.80	1.05	0.21	0.050	NA	NA	NA	NA	NA
70% wood-based biochar (*N* = 2)	0.24	0.32	0.39	0.08	0.439	0.16	0.23	0.30	0.07	0.439	0.09	0.27	0.45	0.18	0.439
30% wood-based biochar (*N* = 2)	0.30	0.54	0.77	0.23	0.121	0.23	0.42	0.60	0.18	0.121	0.14	0.36	0.58	0.22	0.439
Sand filter (*N* = 5**)	0.18	0.50	0.94	0.27	0.251	0.09	0.35	0.60	0.21	0.347	0.27	0.46	0.65	0.19	0.121

(C) Full-scale biochar filtration at the equine college																				
Tertiary treatment	*E. coli* (log_10_)					Intestinal enterococci (log_10_)					*C. perfringens* (log_10_)					Somatic coliphages (log_10_)				
	Min.	Mean	Max.	SD	*p*	Min.	Mean	Max.	SD	*p*	Min.	Mean	Max.	SD	*p*	Min.	Mean	Max.	SD	*p*
Biochar filter (*N* = 3)	−0.13	0.12	0.54	0.30	0.827	−0.98	−0.14	0.41	0.60	0.827	0.00	0.83	1.48	0.62	0.369	−1.00	−0.10	0.60	0.67	1.000
Sand filter (*N* = 3)	−0.08	−0.05	0.00	0.04	0.658	−1.14	−0.45	−0.08	0.49	0.127	−0.20	0.42	1.48	0.75	0.658	−0.40	−0.07	0.20	0.25	1.000

Fecal microbe numbers before and after treatment are presented in the Supplementary Table S10. *N*, Number of treated samples. *For intestinal enterococci, *N* = 5. **For somatic coliphages, *N* = 2. NA, not analyzed. The Kruskal–Wallis test was used to determine the *p*-value from the number of microbes before and after treatment (Supplementary Table S10).

### 3.3 Quantitative microbial risk assessment

**Figure 6:**
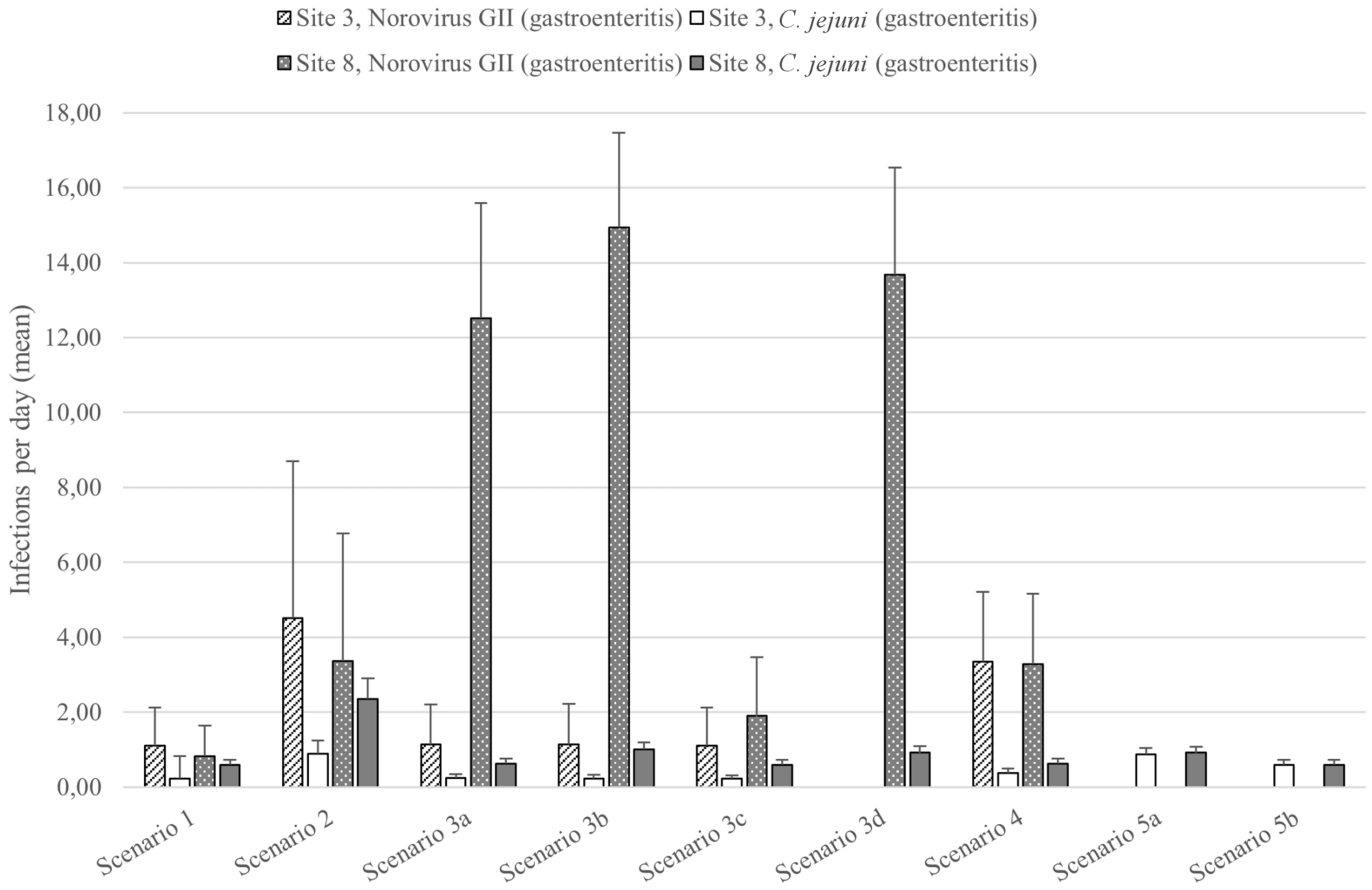
The mean numbers and standard deviations of the gastroenteritis cases caused by norovirus GII and *C. jejuni* at sites 3 and 8 during QMRA scenarios. See Table 1 for descriptions of the scenarios.

The pathogen numbers detected at sites 3 and 8 were used in the BAU scenario for the QMRA (Table 2). At site 3, the mean number of gastroenteritis cases caused by norovirus GII was 1.1 and that caused by *C. jejuni* was 0.2 infections per day among the 50 daily beach goers in the BAU situation (Figure 6). At site 8, the mean number of gastroenteritis cases caused by norovirus GII and *C. jejuni* was 0.8 and 0.6 infections per day, respectively, when the population exposed was 50 people per day.

When the number of visitors was increased to 200 visitors per day during the heatwave scenario (scenario 2), the number of infections per day increased to 4.5 gastroenteritis cases caused by norovirus GII and 0.9 cases caused by *C. jejuni*, therefore being the scenario with highest number of bathers infected at site 3 (Figure 6). The heatwave scenario also caused the highest number of *C. jejuni* cases (2.4 cases per day) at site 8.

As the microbial pollution increased by 50% (scenario 3a), the norovirus and *C. jejuni* infections per day at site 3 increased to 1.2 and 0.3, respectively (Figure 6). After wetland treatment (scenario 3b), the number of *C. jejuni* infections was 0.2, while the number of norovirus GII infections did not change. UV-LED disinfection (scenario 3c) reduced the number of gastroenteritis cases to the same level as in the BAU situation.

The effects of the wastewater contamination scenarios were generally greater at site 8 and caused the highest number of norovirus GII gastroenteritis cases (1.9–14.9 cases per day) (Figure 6). At site 8, the wetland treatment was in use in the BAU situation. When the wastewater contamination occurred together with the absence of wetland treatment in scenario 3b, the highest number of campylobacteriosis cases at the site 8 was caused (1.0 per day). Only slightly lower number of cases (0.9 per day) was caused, when the wetland treatment was omitted without the increase in the microbe numbers present in the wastewater effluent (scenario 3d).

At site 3, contamination from urban runoff caused 3.4 norovirus GII infections per day (Figure 6). The number of *C. jejuni* cases was relatively low, being 0.4 per day. At site 8, the number of norovirus GII infections (3.3 per day) caused by contamination from urban runoff was relatively low when compared to the wastewater contamination scenarios (Figure 6). The number of *C. jejuni* infections was 0.6 per day.

In the animal farm runoff scenario (scenario 5a), the *C. jejuni* infections increased to 0.9 gastroenteritis cases per day at sites 3 and 8 (Figure 6). Biochar filtration reduced the number of *C. jejuni* infections to 0.6 gastroenteritis cases per day. Therefore, animal farm runoff caused the highest *C. jejuni* infection risk of the scenarios considered at site 3.

## 4 Discussion

### 4.1 Bathing water quality and pathogen transport in Lake Vanajavesi and Lake Ormajärvi

In this study, all the analyzed pathogenic microbes were detected at least once from the WWTP discharge areas, and indicator microbe numbers were higher in the discharge areas than other surface water sampling sites. Therefore, this study further demonstrates that primary and secondary wastewater treatment is not efficient enough for pathogenic microbe removal, as already stated in previous studies (Koivunen et al., 2003; Pradhan et al., 2013). For QMRA, the MST marker HF183 was used to model the dilution of the fecal microbes in the lake water. Less of the marker was found from the sampling sites further from the WWTP discharge areas, HF183 DNA being below LOD in majority of the bathing water samples. Moreover, the indicator microbe numbers indicated excellent bathing water quality at site 3 based on the results of four earlier bathing seasons (European Parliament and Council, 2006). In this study, indicator microbe results were below limit values for single sample set by the Ministry of Social Affairs and Health Finland Decree 177/2008 (2008) and Ministry of Social Affairs and Health Finland Decree 354/2008 (2008) for inland waters (*E. coli*: 1000 cfu/mpn/100 ml; intestinal enterococci: 400 cfu/mpn/100 ml) at sites 3 and 8, as well indicating good or excellent bathing water quality. However, the pathogenic microbes, *C. jejuni*, *C. lari*, *Salmonella*, and adenovirus, were detected from bathing water during single sampling events in this study. *Campylobacter* spp. and adenovirus have previously been detected from bathing sites in Finland (Hokajärvi et al., 2013), and they, in addition to *Salmonella*, are not uncommon findings in European bathing waters (Obiri-Danso and Jones, 1999; Wyn-Jones et al., 2011; Schippmann et al., 2013). Higher fecal microbe numbers were detected from the water samples during cooler and rainier periods in this study, which is in line with previous findings (Bradford et al., 2013; Guzman Herrador et al., 2016; Korajkic et al., 2019).

The indicator microbe numbers detected in this study from the runoff discharge areas were lower than the microbe numbers in the WWTP discharge areas, but some indicator microbe observations clearly exceeded the bathing water hygienic quality limit values for single sample set by the Ministry of Social Affairs and Health Finland Decree 177/2008 (n.d.) for inland waters. This is in line with the observations of previous studies (Chong et al., 2013; Paule-Mercado et al., 2016) and confirms that runoff waters may carry significant amounts of fecal pollution to surface waters. However, the number of observations at sites 10, 12, and 13 was low, and the results regarding contamination from urban runoff are therefore not comprehensive.

*Campylobacter jejuni* and *Salmonella* were detected in runoff waters from the horse farm in this study. In addition, some fecal indicator microbe observations exceeded those in the WWTP discharge areas. Runoff waters from horse farms have not been comprehensively investigated, and research has mostly focused on nutrients in the runoff waters (Parvage et al., 2015). However, those studies focusing on fecal microbes have reported relatively high numbers of fecal indicator microbes (Airaksinen et al., 2007; Uusi-Kämppä et al., 2012). Our study suggests that runoff waters from horse farms might be important sources of fecal microbes and pathogens. However, the horse-specific MST marker was only detected once in runoff water from the riding school, but not from the equine college. This suggests that the source of fecal pathogens detected at the equine college might not have been horses. It is also possible that the HorseCytB marker sensitivity is not high enough for water samples, as it was only detected from site 17, where the number of fecal indicator microbes exceeded the numbers measured at WWTP discharge areas. Therefore, HorseCytB marker is not alone sufficient for evaluating contamination from horse farms but should be used together with fecal indicator microbe analyses or other MST markers. In general, the number observations from the water samples from horse farms was small and therefore not comprehensive. In this study, the general sample number was small, being 10 or below replicates from every target area and risk management method test. The low sample number makes the study more susceptible to errors and therefore affects the statistical reliability. This may have led to under or overestimation of the hygienic quality of the water samples studied.

### 4.2 Gastroenteritis risk for bathers

According to the QMRA results, norovirus GII and *C. jejuni* caused a gastroenteritis risk for bathers in every scenario studied, when the visitor number was 50 or 200 during the heatwave scenario. However, the gastroenteritis risk in the BAU situation was very small due to the low number of pathogenic microbes in water. Norovirus caused a greater gastroenteritis risk compared to *C. jejuni*. This is explained by the smaller infectious dose of norovirus (Teunis et al., 2005, 2008) and more frequent detection from WWTP discharge areas. In case of low pathogen numbers at the exposure sites, the MST marker HF183 could be used as a pathogen dilution coefficient for the watershed under evaluation. However, the fate of the different microbes in the watershed might differ through UV radiation, temperature, predation, etc., which should be considered.

When the visitor number increased by 300% during the heatwave scenario, the number of infections also increased by approximately 300%. Exceptionally warm weather may cause an increase in visitor numbers, with more people therefore being exposed to the pathogens present in the water (Schets et al., 2011; Kauppinen et al., 2017). However, only the microbe numbers detected at the bathing site in the BAU situation were considered in the scenario. In reality, the number of microbes in the water may also increase in connection with increased visitor numbers due, for example, to increased secretion of pathogenic microbes into the water or the mixing of contaminated bottom sediment or sand into the water (Elmir et al., 2009; Fewtrell and Kay, 2015). Therefore, a higher visitor frequency may cause in addition to the increased total number of infections, also an increase in the gastroenteritis risk. However, it does not effect on the individual risk of infection.

During different wastewater contamination scenarios, the number of norovirus GII cases was 1–15 per day, and the number of *C. jejuni* cases was one per day. Wastewater contamination of surface waters is one of the most studied threats to bather health. In QMRA studies, norovirus has been evaluated to be one of the most prominent etiological agents causing gastrointestinal (GI) tract infections in bathers exposed to fecally contaminated recreational waters (Soller et al., 2010a,b). Soller et al. (2010a) estimated norovirus from secondary treated wastewater to cause 50 infections among 1,000 visitors at the bathing site, whereas *C. jejuni* caused four infections. However, dilution of the pathogens was not considered in the study by Soller et al. (2010a). The present study was mostly conducted during the COVID-19 pandemic restrictions (summer and autumn 2020), which, through decreased infection frequency (Kuitunen et al., 2022; Liu et al., 2022), probably affected the pathogen numbers present in wastewater. For example, the number of norovirus cases in 2020 was only 31%, and the number of campylobacteriosis case was 45% of the case number in the previous five years (Finnish Institute for Health and Welfare, 2023).

The urban runoff scenario caused three norovirus GII cases and less than one *C. jejuni* case per day. Precipitation associated runoff waters are known to contain high numbers of fecal microbes (Jiang et al., 2015; Paule-Mercado et al., 2016) and therefore cause a possible risk of pathogen spread into surface waters. Pet and bird droppings, in addition to sewer misconnections and overflows, cause significant fecal pollution of urban surface waters (Paule-Mercado et al., 2016; Staley et al., 2016; Steele et al., 2018), and the pathogen sources can therefore either be humans or animals.

According to the scenario 5a, the animal farm runoff caused approximately one *C. jejuni* case per day. Many animal species, such as poultry, cattle, and wildlife, are known *Campylobacter* spp. reservoirs. Occurrence of *Campylobacter* spp. has been also reported from healthy horses (Moriarty et al., 2015; Paruch and Paruch, 2022). Animal farm runoff, manure spread on fields, and wildlife droppings can possess a risk of campylobacteriosis in humans through surface waters (Mulder et al., 2020). Runoff waters from horse farms and pastures may contain significant numbers of fecal microbes and increase the risks of zoonotic waterborne epidemics (Airaksinen et al., 2007; Uusi-Kämppä et al., 2012; Paruch et al., 2020), but number of QMRA studies on the impact of horse farms on water safety is scarce. Previous studies have determined cattle fecal contamination to cause approximately as high an infection risk as wastewater contamination (48 cases per 1,000 bathers), when runoff waters from fields after manure spreading were investigated (Soller et al., 2015). Fecal contamination from swine and poultry causes a smaller, but significant infection risk (Soller et al., 2015). Similar results were obtained in a study using literature-reported pathogen prevalence figures in farmed animals in a QMRA model (Soller et al., 2010b).

### 4.3 Effect of wetland treatment, UV-LED disinfection, and biochar filtration on the risk of infection

The hygienic quality of wastewater effluent can be increased by various tertiary treatments (Pradhan et al., 2013; Wu et al., 2016; Bhatt et al., 2020). QMRA calculations in this study demonstrated that wetland and UV-LED treatment of wastewater effluents could decrease the infection risk at bathing sites below WWTP discharge areas. Wetland treatment with a 40-day water residence time decreased the number of *Campylobacter* spp. by 0.5 log_10_ and norovirus GII by 1.0 log_10_. Previous studies have reported varying norovirus GII removal efficiencies, as the removal efficiencies of enteric viruses varied from 1 to 3 log_10_ (Rachmadi et al., 2016). Furthermore, 2–3.5 log_10_ fecal indicator removal efficiencies have been reported (Stefanakis et al., 2019). However, it has been shown, that bird activity at constructed wetlands may even increase the *Campylobacter* spp. numbers during the wetland treatment (McMinn et al., 2019). In QMRA, wetland treatment reduced the number of norovirus GII cases by 0–2 infections per day and *C. jejuni* cases by 0–0.5 per day during wastewater contamination scenarios. At site 8, where wetland treatment was in use in the BAU situation, bypassing the wetland treatment caused an increase of 13 norovirus GII cases per day. To our knowledge, the effect of wastewater wetland treatment on bathing water safety has not previously been investigated.

In case that UV-LED disinfection could be utilized in full-scale applications by using 10.4 mJ/cm^3^ dose, it could reduce the norovirus GII cases by 0–11 cases during wastewater contamination scenarios. In this study, the impact on the *C. jejuni* cases per day was minimal, likely through small increase of the *C. jejuni* cases during wastewater contamination scenarios. In previous studies, disinfection by UV alone has resulted *E. coli* removal efficiency of around 2.0–3.0 log_10_ and coliphage removal efficiency of around 1.0 log_10_ from ultra-pure and tap water (Fang et al., 2014; Qi et al., 2020). Hokajärvi et al. (2018) found UV disinfection to remove 3.5 log_10_ of *E. coli* and 1.5 log_10_ of F-Specific coliphages during raw water treatment processes. Petterson et al. (2021) estimated the elimination capacity of UV disinfection to be 5.0 log_10_ for bacteria and 2.6 log_10_ for viruses in chemically disinfected swimming ponds. The qualities of the treated wastewater, such as turbidity and organic matter in the water, affect the efficiency of the UV-LED treatment (Zhang et al., 2023), and in large water volumes (i.e., in full-scale applications), their impact can be even bigger in comparison to the pilot scale presented in this study.

Biochar filtration was observed to reduce the number of indicator microbes in treated wastewater by 0.2–0.9 log_10_. However, the number of replicates used in the pilot study was low, which may affect the reliability of the model. In case that 50% wood-based biochar and 50% sand filter with an *E. coli* removal efficiency of 0.6 log_10_ was utilized in on-site applications, it could reduce the *C. jejuni* cases by 0.3 infections per day during animal farm runoff scenarios. The wood-based biochar filter was selected since the most efficient filter type constructed of 50% sludge-based biochar and 50% sand (0.9 log_10_ *E. coli* removal) was noticed to release phosphorus into the treated water. Phosphorus release from sludge-based and biomass-based biochar has also been observed before (Cui et al., 2016; Zhang et al., 2022). On-site biochar filters have previously been tested on farms, but mainly for irrigation water treatment, resulting in 1.0–3.9 log_10_ indicator microbe removal (Kaetzl et al., 2019; Perez-Mercado et al., 2019). In our study, biochar filters were also in use at the horse farms, but their removal efficiencies were observed to be very low or non-existent. Under field conditions, multiple factors, such as filter freezing, or blockage may affect the filtration efficiency. Additionally, the horse-specific MST marker was not detected before (site 14) or after (site 15) the biochar filters at the equine college, which might indicate that the fecal microbes did not originate from horses. For example, wild birds might be the source of fecal microbes on both sides of the filters, which make it difficult to evaluate the filtration efficiency.

The pathogen removal efficiencies with UV-LED disinfection and biochar filtration were estimates based on fecal indicator microbe removal efficiencies, as the effects on norovirus GII and *C. jejuni* were not directly investigated. Therefore, comprehensive studies on the enteric virus and *Campylobacter* spp. removal efficiencies of wastewater tertiary treatment techniques are needed.

## 5 Conclusion

Intestinal microbes were detected together with the human fecal marker HF183 from the discharge areas of WWTPs to the bathing sites, indicating the WWTPs to be the important pollution sources at the bathing sites. The horse-specific marker HorseCytB was detected only once from the horse farm runoff waters.

- Herein, the decrease of MST marker HF183 was used to evaluate how microbes from wastewater were diluted into the receiving watershed. Such approach could be used also elsewhere where microbial transport models are not available to support the risk assessment, although also other factors than dilution such as effect of UV radiation from sunlight might cause decrease of human-specific molecular markers in watersheds.
- Based on the association between fecal microbe numbers present in the water samples and the weather conditions prior to the sampling, the authors conclude that for comprehensive understanding of bathing water hygienic quality, samples during different weather conditions need to be taken.
- Norovirus GII infection risk was especially increased during the wastewater contamination scenarios, whereas *C. jejuni* infection risk was associated with contamination from animal farm runoff.
- Based on indicator microbe numbers, the tertiary wetland treatment and the UV-LED disinfection of wastewater reduced the gastroenteritis risk during the wastewater contamination scenarios.
- QMRA-based risk assessment was successfully used for health risk evaluations at bathing sites in this study, and the risk assessment was supported by scenario modeling including studies on fecal contamination sources, fecal microbe transport in watersheds, and the efficiency of tertiary treatment for fecal pathogens.

## Data availability statement

The raw data supporting the conclusions of this article will be made available by the authors, without undue reservation.

## Author contributions

AR: Conceptualization, Data curation, Formal analysis, Methodology, Visualization, Writing – original draft, Writing – review & editing. PM: Conceptualization, Data curation, Formal analysis, Methodology, Software, Writing – review & editing. KV: Conceptualization, Data curation, Formal analysis, Investigation, Methodology, Writing – review & editing. A-MH: Conceptualization, Investigation, Methodology, Project administration, Writing – review & editing. JR: Conceptualization, Investigation, Methodology, Project administration, Writing – review & editing. JN: Conceptualization, Data curation, Investigation, Methodology, Writing – review & editing. HM: Conceptualization, Funding acquisition, Project administration, Resources, Writing – review & editing. TT: Conceptualization, Funding acquisition, Project administration, Resources, Writing – review & editing. RK: Writing – review & editing. TP: Conceptualization, Funding acquisition, Project administration, Resources, Supervision, Writing – review & editing.
