# Supplementary material for: Scenario-based assessment of fecal pathogen sources affecting bathing water quality: novel treatment options to reduce norovirus and Campylobacter infection risks
Source: Front Microbiol. 2024 Apr 2;15:1353798. doi: 10.3389/fmicb.2024.1353798 (PMC11018956; doi:10.3389/fmicb.2024.1353798)
Supplement: Supplementary file 1 [file Data_Sheet_1.docx]

Supplementary Material

Scenario-based assessment of fecal pathogen sources affecting bathing water quality: Novel treatment options to reduce norovirus and *Campylobacter* infection risks

Annastiina Rytkönen^*^, Päivi Meriläinen, Kristiina Valkama, Anna-Maria Hokajärvi, Josefiina Ruponen, Jarkko Nummela, Harri Mattila, Tiina Tulonen, Rauni Kivistö, Tarja Pitkänen

*** Correspondence:** Annastiina Rytkönen; annastiina.rytkonen@helsinki.fi

# Supplementary Material 1: Sampling sites

## Vanajavesi sampling area

The Paroinen wastewater treatment plant (WWTP) is the wastewater contamination source in Lake Vanajavesi area. Paroinen WWTP is operated by Hämeenlinnan Seudun Vesi Ltd (HS-Vesi), and it treats approximately 17 000 m^3^ wastewater per day. The Paroinen WWTP is a secondary treatment plant using an activated sludge process in wastewater treatment. The WWTP has a two-stage biological treatment system with organic matter removal and denitrification in the first process. In the second process, ammonium is oxidized to nitrate and recirculated to the first process. The majority of phosphorus is removed with iron sulfate precipitation. After biological treatment, phosphorus removal is finalized with dissolved air flotation using aluminiumsulfate as a precipitant before discharging treated wastewater to Lake Vanajavesi.

Site 3 is an EU bathing area in Lake Vanajavesi with approximately 50 visitors per day during the bathing season (June 15th to August 31st). It is a sandy beach with a dock, a life buoy, a notice board, toys, toilets and changing rooms. According to the bathing site profile of site 3, the main pollution sources at the bathing site are sewer networks, runoff waters, agriculture, industry, and boat, car, and train traffic. The water quality at the site was classified as excellent (EC 2006) during the bathing seasons 2017-2020.

Site 4 is a small public bathing area with a visitor frequency of less than 50 per day. Changing rooms, toilets, and a dock are located on the bathing site. Sampling site 5 was a control site upstream the discharge area describing the general microbial contamination in the watershed.

## Ormajärvi sampling area

The Lammi WWTP is the main wastewater contamination source in Lake Ormajärvi area. It belongs to HS-Vesi and treats approximately 1 000 m^3^ wastewater per day. The Lammi WWTP has an activated sludge treatment, the effectiveness of which is increased with biological and chemical treatments. In addition, two final sedimentation ponds and a constructed wetland pond are in use as tertiary treatment. The size of the wetland pond is 40 000 m^2^ and the water residence time is 40 days (Uusheimo *et al.*, 2018). Sampling site 6 was the wastewater outlet before the constructed wetland, and sampling site 7 the discharge site from the wetland to Lake Ormajärvi.

Site 8 is a small public bathing area in Lake Ormajärvi with approximately 50 visitors per day during the bathing season. Changing rooms, toilets, a fire pit site and beach volley courts are located at the bathing site. As there is no requirement for a bathing water profile for small public bathing areas (Ministry of Social Affairs and Health Finland, 354/2008), the profile has not been determined for site 8 to evaluate the pollution sources. However, it is located very close (approximately 500 meters) to site 7 discharge area.

Site 9 is a small public bathing area located on the opposite side of Lake Ormajärvi, approximately 3 500 m from site 7 discharge area. The visitor frequency is similar to site 8, approximately 50 visitors per day during bathing season. Changing rooms, toilets, and a beach volley court are located on the bathing site.

## Runoff water sampling sites

The urban runoff water samples were collected from one stream in suburban (sites 10 and 11) and two streams in urban (sites 12 and 13) areas in the Kanta-Häme region. Site 10 was a control sampling site before the suburban runoff discharge area.

Samples to investigate the microbes in animal farm runoff waters were taken from an equine college with approximately 250 horses (sites 14-16) and from a smaller riding school with approximately 50 horses (sites 17-18). The samples from the equine college were taken from a ditch collecting runoff water from the farm before (site 14) and after biochar and sand filtration (site 15). A sand filter was in use as a control (site 16). The samples from the riding school were collected from header drainpipes installed in ground layers filled with biochar and sand (site 17) collecting runoff from the farm. The site was located near paddocks on the farm. Samples were also taken from the shallow water of the lake next to the riding school (site 18).

**Supplementary Table S1.** Weather observations at A) Lake Vanajavesi area and B) Lake Ormajärvi area during the sampling events. Air temperatures (°C) and precipitation (mm) during the sampling events are presented together with 7- and 30-day mean air temperatures and with precipitation during 3, 7 and 30 days prior to sampling (Finnish Meteorological Institute, 2023).

| 1. Lake Vanajavesi area | | | | | | | |
| --- | --- | --- | --- | --- | --- | --- | --- |
| Sampling dates | Air temperature during sampling (°C)* | 7-day mean air temperature (°C) | 30-day mean air temperature (°C) | Precipitation on the sampling day (mm) | Cumulative precipitation | | |
|  |  |  |  |  | Previous 3 days (mm) | Previous 7 days (mm) | Previous 30 days (mm) |
| 19.11.2019 | 5 | 3 | 2 | 0.4 | 3.1 | 22.7 | 60.9 |
| 8.6.2020 | 14 | 14 | 10 | 0.0 | 12.4 | 17.2 | 37.9 |
| 9.6.2020 | 13 | 14 | 11 | 2.1 | 7.4 | 19.3 | 40.0 |
| 29.6.2020 | 23 | 22 | 18 | 0.0 | 0.0 | 0.0 | 28.4 |
| 30.6.2020 | 15 | 22 | 18 | 7.3 | 7.3 | 7.3 | 35.7 |
| 2.7.2020 | 16 | 20 | 18 | 0.7 | 24.2 | 24.2 | 52.6 |
| 27.7.2020 | 23 | 15 | 16 | 0.0 | 2.9 | 17.3 | 109.8 |
| 28.7.2020 | 17 | 15 | 16 | 8.8 | 9.2 | 26.1 | 118.6 |
| 17.8.2020 | 20 | 16 | 17 | 0.0 | 0.0 | 1.2 | 49.2 |
| 18.8.2020 | 17 (site 3)  20 (site 4) | 16 | 17 | 0.0 | 0.0 | 1.2 | 49.2 |
| 15.9.2020 | 14 (site 3)  15 (site 4) | 11 | 14 | 0.0 | 10.5 | 37.2 | 63.8 |
| 1. Lake Ormajärvi area | | | | | | | |
| Sampling dates | Air temperature during the sampling (°C)* | 7-day mean air temperature (°C) | 30-day mean air temperature (°C) | Precipitation on the sampling day (mm) | Cumulative precipitation | | |
|  |  |  |  |  | Previous 3 days (mm) | Previous 7 days (mm) | Previous 30 days (mm) |
| 9.6.2020 | 13 | 13 | 10 | 8.1 | 14.1 | 21.3 | 54.7 |
| 29.6.2020 | 19 | 21 | 17 | 0.0 | 0.0 | 0.0 | 40.2 |
| 30.6.2020 | 16 | 21 | 17 | 1.4 | 1.4 | 1.4 | 41.6 |
| 2.7.2020 | 13 | 19 | 17 | 7.0 | 40.0 | 40.0 | 80.2 |
| 27.7.2020 | 17 | 15 | 15 | 0.0 | 14.3 | 26.1 | 139.5 |
| 28.7.2020 | 16 | 15 | 15 | 11.4 | 11.9 | 37.5 | 150.9 |
| 17.8.2020 | 15 | 15 | 16 | 0.0 | 0.0 | 0.0 | 53.4 |
| 18.8.2020 | 14 | 15 | 16 | 0.0 | 0.0 | 0.0 | 53.4 |

* Air temperatures during the sampling events were collected real time from the Finnish Meteorological Institute mobile application.

**Supplementary Table S2.** The numbers of water samples collected at sites 1-18 for each microbial analysis conducted in the study. Total sample numbers and the microbes analyzed from the sampling sites in Lake Vanajavesi (sites 1-5), Lake Ormajärvi (sites 6-9), runoff water (sites 10-13) and horse farms (sites 14-18). Intestinal pathogens, fecal indicator microbes, and human (HF183) and horse (HorseCytB) specific QMST markers were analyzed from the water samples.

| **Microbial analyses** | **Lake Vanajavesi sampling area** | | | | | **Lake Ormajärvi sampling area** | | | | **Runoff water sites** | | | | **Horse farms** | | | | |
| --- | --- | --- | --- | --- | --- | --- | --- | --- | --- | --- | --- | --- | --- | --- | --- | --- | --- | --- |
|  | **Site 1 WW** | **Site 2 D** | **Site 3 B** | **Site 4 B** | **Site 5 C** | **Site 6 WW** | **Site 7 WWW** | **Site 8 B** | **Site 9 B** | **Site 10** | **Site 11** | **Site 12** | **Site 13** | **Site 14** | **Site 15** | **Site 16** | **Site 17** | **Site 18** |
| Thermotolerant *Campylobacter* spp. | 1 | 1 | 4 | NA | NA | 6 | 6 | 4 | NA | NA | NA | NA | NA | 4 | NA | NA | NA | NA |
| Salmonella | 1 | 1 | 4 | NA | NA | 6 | 6 | 4 | NA | NA | NA | NA | NA | 4 | NA | NA | NA | NA |
| Adenovirus, sapovirus, norovirus | 2 | 1 | 4 | NA | NA | 5 | 5 | 4 | NA | NA | NA | NA | NA | NA | NA | NA | NA | NA |
| *E. coli* and intestinal enterococci | 6 | 6 | 5 | 4 | 3 | 6 | 6 | 5 | 4 | 1 | 7 | 2 | 2 | 6 | 3 | 3 | 3 | 2 |
| Coliform bacteria | 6 | 6 | 4 | 4 | 3 | 6 | 6 | 4 | 4 | NA | 3 | NA | 1 | 4 | 3 | 3 | 2 | 1 |
| Sulfite-reducing clostridia | 6 | 6 | 4 | 4 | 3 | 6 | 6 | 4 | 4 | NA | 3 | NA | NA | 4 | 3 | 3 | 2 | NA |
| *C. perfringens* | 6 | 6 | 4 | 4 | 3 | 6 | 6 | 4 | 4 | NA | 3 | NA | 1 | 4 | 3 | 3 | 1 | NA |
| Somatic and F-specific coliphages | 6 | 6 | 4 | 4 | 3 | 6 | 6 | 4 | 4 | NA | 3 | NA | NA | 4 | 3 | 3 | 1 | NA |
| HF183 marker | 4 | 5 | 4 | 4 | 3 | NA | 4 | 4 | 4 | NA | 3 | NA | NA | 4 | NA | NA | NA | NA |
| HorseCytB marker | NA | 5 | 4 | 4 | 3 | NA | NA | NA | NA | NA | NA | NA | NA | 4 | NA | NA | 1 | 1 |
| **Total number of samples** | **7** | **7** | **5** | **4** | **3** | **10** | **10** | **5** | **4** | **1** | **7** | **2** | **2** | **6** | **3** | **3** | **3** | **2** |

WW = Wastewater discharge. D = Sampling site downstream from the discharge area. B = Bathing site. C = Control site. WWW = Wetland treated wastewater discharge. NA = Not analyzed.

# Supplementary Material 2: Quantitative PCR analyses

Adenoviruses, noroviruses (genotypes I and II), sapoviruses, and microbial source tracking (MST) markers were determined from the samples by qPCR. To control the concentration methods and nucleic acid extraction methods, the negative concentration controls, filtration controls and negative extraction controls with extraction reagents only were processed alongside the samples.

The adenovirus, norovirus and sapovirus analyses were carried out using ten-fold serial dilutions of gBlocks Gene Fragments (Integrated DNA Technologies, United States), generated using reference sequences of the target sequences, were run with every assay: 5^1^, 10^1^, 10^2^, 10^3^, 10^4^, 10^5^ and 10^6^ copies/rxn. The reference sequences selected by using the NCBI Nucleotide BLAST program (National Center for Biotechnology Information, United States National Library of Medicine) are presented in the Table S3. No template control (NTC) including reverse transcription and PCR reagents and HyClone Water (GE Healthcare, Life Sciences, United Kingdom) was run with every standard set. Three parallel samples were run from undiluted and 10-fold diluted samples. One of the parallel samples was used to control inhibition in reverse transcription and PCR reactions by external amplification control plasmid standard (ISO 15216-1:2017). If inhibition was detected, the diluted samples were used for final data generation. Sample amount and dilution events from extraction and qPCR reaction were acknowledged in final data generation. If the NA values were below the limit of quantification (LOQ), the result was treated as a present, but not quantitative, and therefore the value was set to half of the (0.5×) LOQ. All normal amplification before the cycle threshold (Ct) value 40 was considered as present in the viral analyses.

The MST markers HF183 and HorseCytB were analyzed as presented in detail by Rytkönen *et al.* (2021). The HF183 marker was determined from DNA and RNA aliquots. As the RNA was converted to cDNA, the possible effect of reverse transcription inhibitors was controlled by performing the cDNA synthesis using 8 µl as undiluted and 0.8 µl as 10-fold dilution of the total RNA. As the HorseCytB marker targets mitochondrial DNA, only DNA aliquot was analyzed. Ten-fold serial dilutions of gBlocks Gene Fragments, generated using reference sequences of the target sequences, were run with every assay: 10^0^, 2 × 10^1^, 2 × 10^2^, 2 × 10^3^, 2 × 10^4^, and 10^5^ copies/µl. NTC was run in duplicate with every standard set. The reference sequences selected by using the NCBI Nucleotide BLAST program are presented in the Table S3. Undiluted and 1:10 and 1:100 diluted DNA and cDNA preparations in HyClone Water were used to detect PCR inhibition. If inhibition was detected, the diluted samples were used for final data generation. The limit of detection (LOD) was set as three copies per reaction, as suggested by Bustin *et al.* (2009). LOD values were subtracted from all the results to generate the final data for the assay. Sample amount and dilution events from extraction, cDNA synthesis, and qPCR reaction were acknowledged. If the NA values were below the limit of quantification (LOQ), the result was treated as a present, but not quantitative, and therefore the value was set to half of the (0.5×) LOQ.

**Supplementary Table S3**. The qPCR assays used in the study.

| Assay | Primer or probe sequence (5’ to 3’) | Annealing temp. (°C) | gBlocks reference sequence^*^ | Reference |
| --- | --- | --- | --- | --- |
| HF183 | HF183-1: ATCATGAGTTCACATGTCCG | 60 | AB242142.1 | Haugland *et al.*, 2010 |
|  | HfBthetR1: CGTAGGAGTTTGGACCGTGT |  |  |  |
|  | HfBthetP1: 6-FAM-CTGAGAGGAAGGTCCCCCACATTGGA-ZEN/IBFQ |  |  |  |
| HorseCytB | HorseF: AGGAGCAACAGTCATCACGAACCT | 60 | MG761997.1 | Schill & Mathes, 2008 |
|  | HorseR: AAATGTACGACTACCAGGGCTGTG |  |  |  |
|  | HorseP: 6-FAM-ATCGGTACTACCCTCGTCGAGTGAAT-BHQ1 |  |  |  |
| Adenovirus | JTVXF: GGACGCCTCGGAGTACCTGAG | 60 | AC_000008.1 | Jothikumar *et al.*, 2005 |
|  | JTVXR: ACIGTGGGGTTTCTGAACTTGTT |  |  |  |
|  | JTVXP: 6-FAM-CTGGTGCAGTTCGCCCGTGCCA-ZEN/IBFQ |  |  |  |
| Sapovirus | SaV124F: GAYCASGCTCTCGCYACCTAC | 60 | AY237420 | Oka *et al.*, 2006 |
|  | SaV1F: TTGGCCCTCGCCACCTAC |  |  |  |
|  | SaV5F: TTTGAACAAGCTGTGGCATGCTAC |  |  |  |
|  | SaV1245R: CCCTCCATYTCAAACACTA |  |  |  |
|  | SaV124TP: 6-FAM-CCRCCTATRAACCA-MGB-NQF |  |  |  |
|  | SaV5TP: 6-FAM–TGCCACCAATGTACCA-MGB-NQF |  |  |  |
| Norovirus GI | NVGIF: GCYATGTTCCGCTGGATG | 60 | M87661.2 | Kauppinen *et al.*, 2014 |
|  | NVGIR: CCTTAGACGCCATCATCATT |  |  |  |
|  | NVGIP-MGB: VIC-TGGACAGGAGAYCGC-MGB-NFQ |  |  |  |
| Norovirus GII | QNIF2d: ATGTTCAGRTGGATGAGRTTCTCWGA | 60 | AF145896.1 |  |
|  | COG2R: TCGACGCCATCTTCATTCACA |  |  |  |
|  | RING2-TP: 6-FAM-TGGGAGGGCGATCGCAATCT-BHQ1 |  |  |  |

^*^The reference sequences selected by using the NCBI Nucleotide BLAST program (National Center for Biotechnology Information, United States National Library of Medicine). 6-FAM = 6-carboxyfluorescein. ZEN/IBFQ = ZEN-Iowa Black FQ Quencher. BHQ1 = Black Hole Quencher 1. MGB-NQF = Minor Groove Binder Nonfluorescent Quencher. VIC = VIC phosphoramidite fluorophore.

**Supplementary Table S4.** Performance characteristics of the qPCR runs conducted in the study.

| Assay | Range of RNA blanks^*^ | Range of DNA blanks^*^ | Limit of detection | Range of amplification efficiency (%) | Range of R^2^ | Range of quantification (copies) |
| --- | --- | --- | --- | --- | --- | --- |
| HF183 | ND | ND | 3 copies/ rxn^**^ | 101.8-111.4 | 0.976-0.997 | 20-200 000 |
| HorseCytB | - | ND | 3 copies/ rxn^**^ | 88.4 | 0.998 | 20-200 000 |
| Adenovirus | - | ND | Ct <40 | 96.1-116.0 | 0.963-0.999 | 5-5 000 000 |
| Sapovirus | ND | - | Ct <40 | 85.1-118.0 | 0.985-0.996 | 5-5 000 000 |
| Norovirus GI | ND | - | Ct <40 | 91.8-116.4 | 0.969-0.999 | 5-5 000 000 |
| Norovirus GII | ND | - | Ct <40 | 103.6-121.0 | 0.967-0.999 | 5-5 000 000 |

^*^Range of copy numbers per reaction (copies/rxn) in negative control samples. ^**^Limit of detection set according to Bustin *et al.,* 2009. For viral analyses, the limit of detection was set below the cycle threshold value (Ct) 40. ND = Not detected. - = Not applicable.

**Supplementary Table S5.** The experimental set-up for testing the microbial removal efficiency in A) UV-LED disinfection and B) biochar filtration treatment processes.

| 1. UV-LED treatment tests | | | |
| --- | --- | --- | --- |
| Test no. | Secondary treated wastewater effluent | Tertiary treatment | No. of replicates |
| 0 | Site 1 | - | 3 |
|  | Site 6 | - | 3 |
| 1 | Site 1 | UV-LED 300 L/h (dose 10.4 mJ/cm^3^) | 3 |
|  | Site 6 | UV-LED 300 L/h (dose 10.4 mJ/cm^3^) | 3 |
| 2 | Site 1 | UV-LED 600 L/h (dose 5.2 mJ/cm^3^) | 3 |
|  | Site 6 | UV-LED 600 L/h (dose 5.2 mJ/cm^3^) | 3 |
| 1. Biochar filtration tests | | | |
| Test no. | Secondary treated wastewater effluent | Tertiary treatment | No. of replicates |
| 1 | Site 6 | - | 2 |
|  |  | 50% wood-based biochar filter, unused | 2 |
|  |  | 50% sludge-based biochar filter, unused | 2 |
|  |  | Sand filter, unused | 2 |
|  |  | Empty filter | 2 |
| 2 | Site 6 | - | 1 |
|  |  | 50% wood-based biochar filter, autoclaved | 1 |
|  |  | 50% sludge-based biochar filter, autoclaved | 1 |
|  |  | Sand filter, autoclaved | 1 |
|  |  | Empty filter | 1 |
| 3 | Site 6 | - | 1 |
|  |  | 70% wood-based biochar filter, unused | 1 |
|  |  | 30% wood-based biochar filter, unused | 1 |
|  |  | Sand filter, unused | 1 |
|  |  | Empty filter | 1 |
| 4 | Site 6 | - | 1 |
|  |  | 70% wood-based biochar filter, autoclaved | 1 |
|  |  | 30% wood-based biochar filter, autoclaved | 1 |
|  |  | Sand filter, autoclaved | 1 |
|  |  | Empty filter | 1 |

# Supplementary Material 3: Data calculation equations for exposure and health effects assessment

All microbial results were handled in the risk assessment calculations as per 1000 mL. The results below the limit of detection (LOD) or the limit of quantitation (LOQ) were determined with the equations Eq. (1) and Eq. (2), respectively.

Eq. (1)

$$Conversion of the results below LOD for risk assessment \left( \left( \frac{CFU}{1000 mL} \right)or \left( \frac{GC}{1000 mL} \right) \right)=0.5*\left( \left( \frac{1}{volume analyzed \left( mL \right)} \right)*1000 \right)$$

Eq. (2)

$$Conversion of the results below LOQ for risk assessment \left( \left( \frac{GC}{1000 mL} \right) \right)=0.5*\left( \left( \frac{5}{volume analyzed \left( mL \right)} \right)*1000 \right)$$

The increase in the norovirus and *C. jejuni* numbers at sites 3 and 8 in the wastewater contamination scenarios were calculated with Eq. (3).

Eq. (3)

$$Increase in microbe numbers at bathing site in wastewater contamination scenarios \left( \frac{CFU}{1000 mL} or\frac{GC}{1000 mL} \right)$$

$$={10}^{log10 (microbe number at the contamination site)-decrease by dilution-decrease by microbe removal technique}$$

**Supplementary Table S6.** Parameters used in the Bathing Water Guide tool.

| Parameter | Values used in the QMRA tool | | | | | Reference |
| --- | --- | --- | --- | --- | --- | --- |
| Time spent in water, ingested water volume and age distribution of bathing site visitors | Age (years) | Time spent in water (h) | Ingested water volume (L/h) | | Percentage of different age groups among bathing site visitors | DeFlorio-Baker *et al.* 2018 |
|  | 0-4 | 0.5-1.5 | 0.024 | | 10 |  |
|  | 5-9 | 0.75-2 | 0.024 | | 20 |  |
|  | 10-14 | 0.75-2 | 0.027 | | 20 |  |
|  | 15-24 | 0.5-2 | 0.020 | | 20 |  |
|  | 25-64 | 0.5-1.5 | 0.014 | | 20 |  |
|  | 65-79 | 0.5-1.5 | 0.013 | | 8 |  |
|  | 80+ | 0.5-1.5 | 0.013 | | 2 |  |
| Dose-response models for waterborne pathogens | Norovirus | | | *C. jejuni* | | Teunis *et al*. 2005; Teunis *et al.* 2008; WHO 2017 |
|  | 0.04 | | | 0.024 | |  |

**Supplementary Table S7.** Pathogen observations at A) sampling sites in Lake Vanajavesi, B) sampling sites in Lake Ormajärvi and C) the equine college. Samples were collected as grab samples into sterile plastic bottles (bottle) and for viral analyses as concentrates with dead-end ultra filtration (DEUF).

| 1. **Lake Vanajavesi** | | | | | | | | | |
| --- | --- | --- | --- | --- | --- | --- | --- | --- | --- |
| **Sampling site** | **Date** | **Sampling method** | ***Campylobacter* spp.** | | ***Salmonella* spp.** | **Adenovirus (GC/100 mL)** | **Norovirus (GC/100 mL)** | | **Sapovirus (GC/100 mL)** |
|  |  |  | ***C. jejuni*** | ***C. lari*** |  |  | **GI** | **GII** |  |
| Site 1 | 19.11.2019 | Bottle | ≥100 cfu/1000 mL | <1 cfu/2220 mL^*^ | ≥100 cfu/1000 mL | <33.11^**^ | <1.14^**^ | 78 | 279 |
|  |  | DEUF | NA | NA | NA | 91 | 37 | 176 | 195 |
| Site 2 | 19.11.2019 | Bottle | ≥100 cfu/1000 mL | <1 cfu/2220 mL^*^ | 1-10 cfu/1000 mL | NA | NA | NA | NA |
|  |  | DEUF | NA | NA | NA | 2 | <1 GC/2124 mL^*^ | <0.24^**^ | <0.24^**^ |
| Site 3 | 8.6.2020 | Bottle | <1 cfu/2220 mL^*^ | <1 cfu/2220 mL^*^ | <1 cfu/1110 mL^*^ | <1 GC/440 mL^*^ | <1 GC/251 mL^*^ | <1 GC/251 mL^*^ | <1 GC/251 mL^*^ |
|  | 29.6.2020 | Bottle | <1 cfu/2220 mL^*^ | 1-10 cfu/1000 mL | 1-10 cfu/1000 mL | <1 GC/264 mL^*^ | <1 GC/189 mL^*^ | <1 GC/189 mL^*^ | <1 GC/189 mL^*^ |
|  | 27.7.2020 | Bottle | <1 cfu/2220 mL^*^ | <1 cfu/2220 mL^*^ | <1 cfu/1110 mL^*^ | <1 GC/314 mL^*^ | <1 GC/291 mL^*^ | <1 GC/291 mL^*^ | <1 GC/291 mL^*^ |
|  | 17.8.2020 | Bottle | <1 cfu/2220 mL^*^ | <1 cfu/2220 mL^*^ | <1 cfu/1110 mL^*^ | <1 GC/308 mL^*^ | <1 GC/223 mL^*^ | <1 GC/223 mL^*^ | <1 GC/223 mL^*^ |

**Supplementary Table S7. (continued)**

| 1. **Lake Ormajärvi** | | | | | | | | | |
| --- | --- | --- | --- | --- | --- | --- | --- | --- | --- |
| **Sampling site** | **Date** | **Sampling method** | ***Campylobacter* spp.** | | ***Salmonella* spp.** | **Adenovirus (GC/100 mL)**  **GI** | **Norovirus (GC/100 mL)** | | **Sapovirus (GC/100 mL)** |
|  |  |  | ***C.jejuni*** | ***C. lari*** |  |  | **GI** | **GII** |  |
| Site 6 | 19.11.2019 | Bottle | <1 cfu/111 mL^*^ | <1 cfu/111 mL^*^ | <1 cfu/111 mL^*^ | 401 | 80 | 23 186 | <0.35^**^ |
|  |  | DEUF | NA | NA | NA | 353 | 19 | 8 340 | 14 |
|  | 9.6.2020 | Bottle | <1 cfu/111 mL^*^ | <1 cfu/111 mL^*^ | <1 cfu/111 mL^*^ | NA | NA | NA | NA |
|  |  | DEUF | NA | NA | NA | <2.60^**^ | 9 | <1 GC/192 mL^*^ | <1 GC/192 mL^*^ |
|  | 29.6.2020 | Bottle | <1 cfu/111 mL^*^ | <1 cfu/111 mL^*^ | <1 cfu/111 mL^*^ | NA | NA | NA | NA |
|  |  | DEUF | NA | NA | NA | 2.05^**^ | 2.05^**^ | 2.05^**^ | <1 GC/244 mL^*^ |
|  | 27.7.2020 | Bottle | <1 cfu/111 mL^*^ | <1 cfu/111 mL^*^ | <1 cfu/111 mL^*^ | NA | NA | NA | NA |
|  |  | DEUF | NA | NA | NA | <2.82^**^ | 50 | 220 | <1 GC/177 mL^*^ |
|  | 17.8.2020 | Bottle | 10-100 cfu/100 mL | <1 cfu/111 mL^*^ | <1 cfu/111 mL^*^ | NA | NA | NA | NA |
|  | 14.9.2020 | Bottle | <1 cfu/111 mL^*^ | <1 cfu/111 mL^*^ | <1 cfu/111 mL^*^ | NA | NA | NA | NA |
| Site 7 | 19.11.2019 | Bottle | <1 cfu/111 mL^*^ | <1 cfu/111 mL^*^ | <1 cfu/111 mL^*^ | 448 | <2.65^**^ | 6 860 | 469 |
|  |  | DEUF | NA | NA | NA | 503 | 31 | 3 881 | 301 |
|  | 9.6.2020 | Bottle | <1 cfu/111 mL^*^ | <1 cfu/111 mL^*^ | <1 cfu/111 mL^*^ | NA | NA | NA | NA |
|  |  | DEUF | NA | NA | NA | <1 GC/174 mL^*^ | <1 GC/174 mL^*^ | <1 GC/174 mL^*^ | <1 GC/174 mL^*^ |
|  | 29.6.2020 | Bottle | <1 cfu/111 mL^*^ | <1 cfu/111 mL^*^ | <1 cfu/111 mL^*^ | NA | NA | NA | NA |
|  |  | DEUF | NA | NA | NA | <1 GC/188 mL^*^ | <2.66^**^ | <1 GC/188 mL^*^ | <1 GC/188 mL^*^ |
|  | 27.7.2020 | Bottle | <1 cfu/111 mL^*^ | <1 cfu/111 mL^*^ | <1 cfu/111 mL^*^ | NA | NA | NA | NA |
|  |  | DEUF | NA | NA | NA | <1 GC/102 mL^*^ | 2 | <1 GC/102 mL^*^ | <1 GC/102 mL^*^ |
|  | 17.8.2020 | Bottle | <1 cfu/111 mL^*^ | <1 cfu/111 mL^*^ | <1 cfu/111 mL^*^ | NA | NA | NA | NA |
|  | 14.9.2020 | Bottle | <1 cfu/111 mL^*^ | <1 cfu/111 mL^*^ | <1 cfu/111 mL^*^ | NA | NA | NA | NA |
| Site 8 | 9.6.2020 | Bottle | 1-10 cfu/1000 mL | 10-100 cfu/100 mL | <1 cfu/1110 mL^*^ | <1 GC/440 mL^*^ | <1 GC/314 mL^*^ | <1 GC/314 mL^*^ | <1 GC/314 mL^*^ |
|  | 29.6.2020 | Bottle | <1 cfu/2220 mL^*^ | <1 cfu/2220 mL^*^ | <1 cfu/1110 mL^*^ | <1.14^**^ | <1 GC/314 mL^*^ | <1 GC/314 mL^*^ | <1 GC/314 mL^*^ |
|  | 27.7.2020 | Bottle | <1 cfu/2220 mL^*^ | <1 cfu/2220 mL^*^ | <1 cfu/1110 mL^*^ | <1 GC/314 mL^*^ | <1 GC/314 mL^*^ | <1 GC/314 mL^*^ | <1 GC/314 mL^*^ |
|  | 17.8.2020 | Bottle | <1 cfu/2220 mL^*^ | <1 cfu/2220 mL^*^ | <1 cfu/1110 mL^*^ | <1 GC/440 mL^*^ | <1 GC/314 mL^*^ | <1 GC/314 mL^*^ | <1 GC/314 mL^*^ |

**Supplementary Table S7. (continued)**

| 1. **Equine college** | | | | | | | | | |
| --- | --- | --- | --- | --- | --- | --- | --- | --- | --- |
| **Sampling site** | **Date** | **Sampling method** | ***Campylobacter* spp.** | | ***Salmonella* spp.** | **Adenovirus (GC/100 mL)**  **GI** | **Norovirus (GC/100 mL)** | | **Sapovirus (GC/100 mL)** |
|  |  |  | ***C.jejuni*** | ***C. lari*** |  |  | **GI** | **GII** |  |
| Site 14 | 17.8.2020 | Bottle | <1 cfu/222 mL^*^ | <1 cfu/222 mL^*^ | 1-10 cfu/100 mL | NA | NA | NA | NA |
|  | 31.8.2020 | Bottle | <1 cfu/222 mL^*^ | <1 cfu/222 mL^*^ | <1 cfu/111 mL^*^ | NA | NA | NA | NA |
|  | 28.9.2020 | Bottle | <1 cfu/2220 mL^*^ | <1 cfu/2220 mL^*^ | <1 cfu/1110 mL^*^ | NA | NA | NA | NA |
|  | 26.10.2020 | Bottle | 1-10 cfu/1000 mL | <1 cfu/2220 mL^*^ | <1 cfu/1110 mL^*^ | NA | NA | NA | NA |

^*^Below the limit of detection. ^**^Below the limit of quantification. NA = Not analyzed.

**
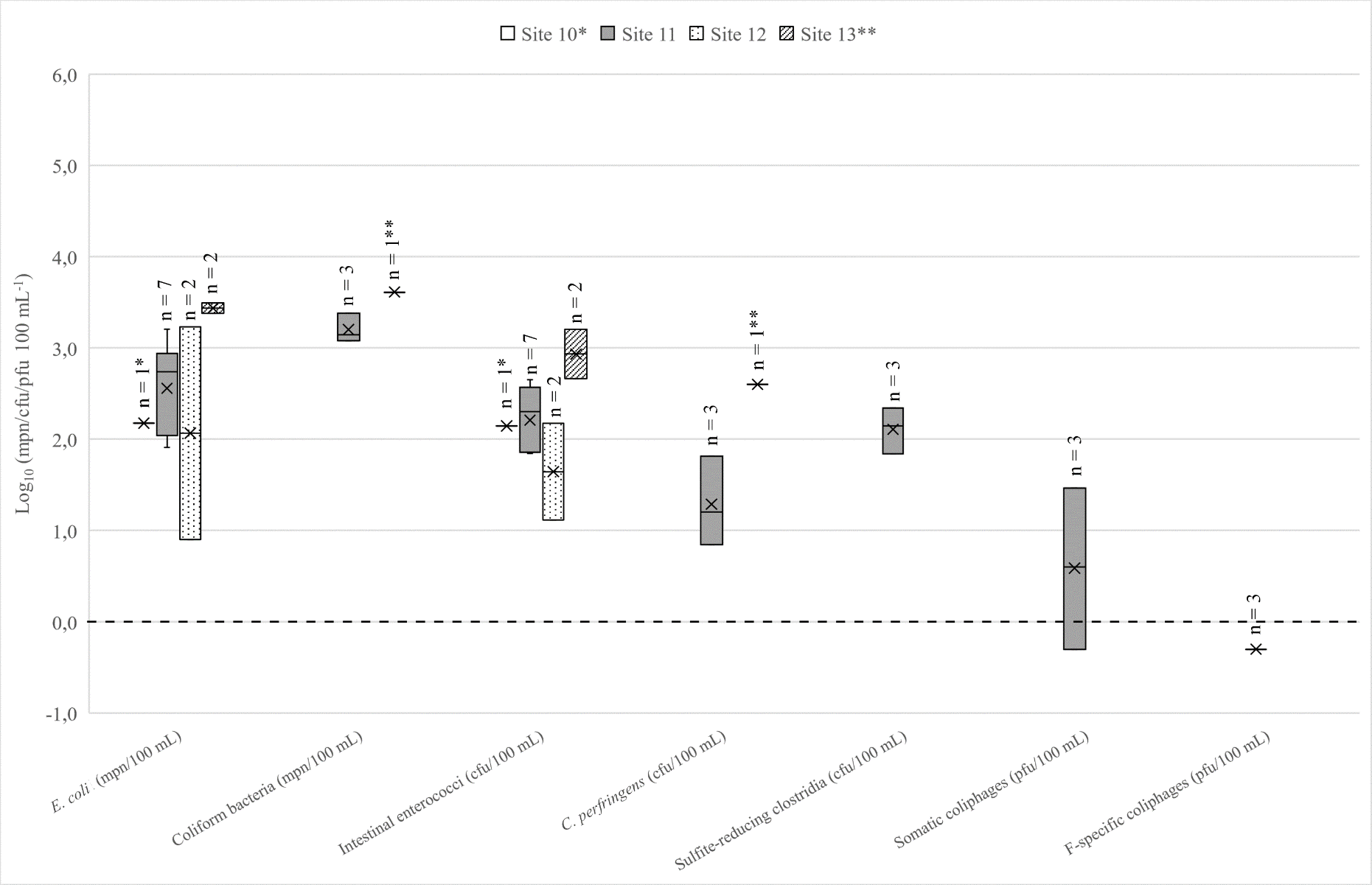
Supplementary Figure S1. Fecal indicator microbes detected in urban runoff waters.** Site 10 = Control site before the suburban runoff discharge site at the site 11. Site 11 = Suburban runoff stream. Site 12 = Urban runoff stream. Site 13 = Urban runoff stream. The mean is presented in the boxplots with a vertical line and the median with a cross. n = Number of the samples analyzed. The limit of detection (LOD) log_10_ 0.0 or 1 mpn/cfu/pfu 100^-1^ is marked as dotted line. The samples below the LOD are presented as 0.5×LOD = log_10_ -0.3 or 0.5 mpn/cfu/pfu 100^-1^. *Site 10. **Site 13.

**
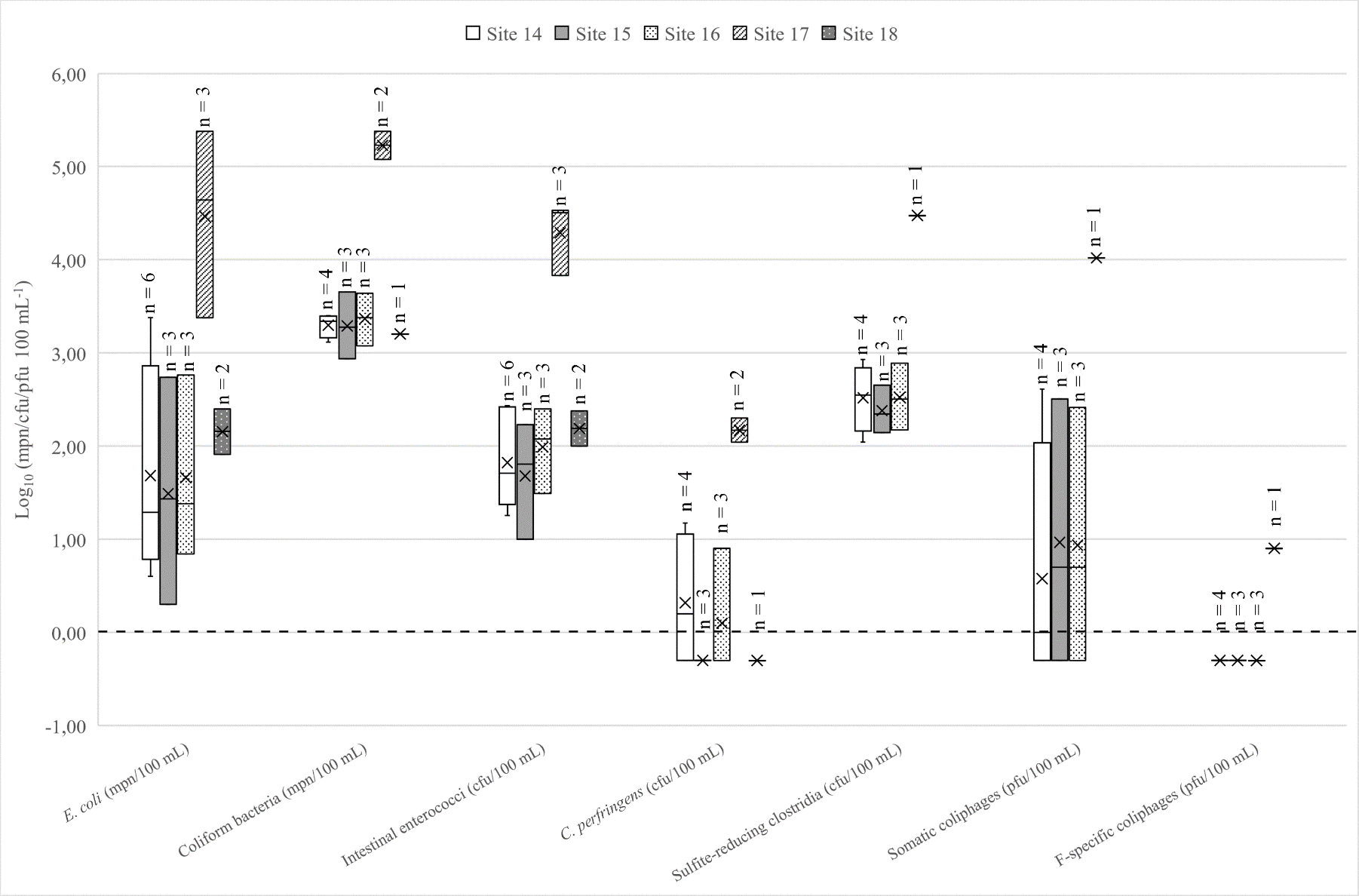
Supplementary Figure S2. Fecal indicator microbes detected in horse farm runoff waters.** Site 14 = Ditch collecting the runoff water from the Equine College. Site 15 = Ditch after biochar and sand filtration collecting the runoff from the Equine College. Site 16 = Ditch after control sand filtration collecting the runoff from the Equine College. Site 17 = Biochar and sand drainpipe collecting the runoff from the riding school. Site 18 = Lake next to the riding school. The mean is presented in the boxplots with a vertical line and the median with a cross. n = Number of the samples analyzed. The limit of detection (LOD) log_10_ 0.0 or 1 mpn/cfu/pfu 100^-1^ is marked as a dotted line. The samples below the LOD are presented as 0.5×LOD = log_10_ -0.3 or 0.5 mpn/cfu/pfu 100^-1^

**Supplementary Table S8.** The relation between air temperature (°C) and precipitation (mm) and the numbers of fecal microbes in the surface water during the sampling periods determined as Spearman’s rank correlation co-efficient (r_s_).

| Parameter |  | Air temperature during the sampling (°C) | 7-day mean air temperature (°C) | 30-day mean air temperature (°C) | Precipitation on the sampling day (mm) | Cumulative precipitation | | |
| --- | --- | --- | --- | --- | --- | --- | --- | --- |
|  |  |  |  |  |  | Previous 3 days (mm) | Prevous 7 days (mm) | Previous 30 days (mm) |
|  | Range | 5-23 | 3-22 | 2-18 | 0.0-11.4 | 0.0-40.0 | 0.0-40.0 | 28.4-150.9 |
|  | Mean | 15.6 | 14.9 | 14.5 | 2.1 | 8.5 | 18.4 | 70.5 |
| *E. coli* | r_s_ | -0.372 | -0.628 | -0.421 | -0.001 | 0.123 | 0.445 | 0.275 |
|  | p-value | 0.033 | <0.001 | 0.015 | 0.997 | 0.494 | 0.010 | 0.121 |
|  | N | 33 | 33 | 33 | 33 | 33 | 33 | 33 |
| Intestinal enterococci | r_s_ | -0.131 | -0.529 | -0.283 | -0.037 | 0.051 | 0.459 | 0.491 |
|  | p-value | 0.466 | 0.002 | 0.111 | 0.837 | 0.779 | 0.007 | 0.004 |
|  | N | 33 | 33 | 33 | 33 | 33 | 33 | 33 |
| *C. jejuni* | r_s_ | -0.740 | -0.877 | -0.630 | 0.570 | 0.430 | 0.799 | 0.450 |
|  | p-value | 0.006 | <0.001 | 0.028 | 0.053 | 0.163 | 0.002 | 0.142 |
|  | N | 12 | 12 | 12 | 12 | 12 | 12 | 12 |
| Somatic coliphages | r_s_ | -0.343 | -0.576 | -0.402 | -0.127 | 0.327 | 0.495 | 0.288 |
|  | p-value | 0.059 | <0.001 | 0.025 | 0.494 | 0.073 | 0.005 | 0.116 |
|  | N | 31 | 31 | 31 | 31 | 31 | 31 | 31 |

N = Number of observations.

**Supplementary Table S9.** The log_10_ minimum, mean and maximum values and standard deviations (SD) of fecal microbes before and after wetland treatment determined by using wastewater samples collected at Lammi WWTP.

| Microbe | Before wetland treatment | | | | After wetland treatment | | | | N |
| --- | --- | --- | --- | --- | --- | --- | --- | --- | --- |
|  | Min. (log_10_) | Mean (log_10_) | Max. (log_10_) | SD (log_10_) | Min. (log_10_) | Mean (log_10_) | Max. (log_10_) | SD (log_10_) |  |
| *E. coli* (mpn/1000 mL) | 3.98 | 4.97 | 5.78 | 0.55 | 0.70 | 2.69 | 4.67 | 1.04 | 11^*^ |
| Intestinal enterococci (cfu/1000 mL) | 3.36 | 4.34 | 4.85 | 0.40 | 1.85 | 2.91 | 4.23 | 0.64 | 12^*^ |
| Sulfite-reducing clostridia (cfu/1000 mL) | 4.38 | 4.79 | 5.21 | 0.25 | 3.26 | 3.72 | 4.32 | 0.42 | 6 |
| *C. perfringens* (cfu/1000 mL) | 4.54 | 4.71 | 4.94 | 0.14 | 2.30 | 3.09 | 4.32 | 0.55 | 6 |
| Somatic coliphages (pfu/1000 mL) | 3.30 | 3.71 | 4.12 | 0.33 | 1.30 | 2.35 | 3.64 | 0.91 | 6 |
| F-specific coliphages (pfu/1000 mL) | 0.70 | 1.81 | 3.82 | 1.06 | 0.70 | 1.19 | 3.64 | 1.10 | 6 |
| Norovirus GII (GC/1000 mL) | 0.42 | 2.66 | 5.37 | 1.59 | 0.42 | 1.73 | 4.84 | 1.89 | 8^*^ |
| *C. jejuni* (cfu/1000 mL) | 0.35 | 1.44 | 3.70 | 1.07 | 0.65 | 1.01 | 1.82 | 0.46 | 10^*^ |

N = Number of before and after treatment pairs analyzed. Results below LOD and LOQ determined with equations Eq. (1) and Eq. (2), respectively (Supplementary Material 2). *Sample pairs collected from the Lammi WWTP (before and after wetland treatment) in 2018 were used in removal efficiency calculations for *E. coli* (5 pairs), enterococci (6 pairs), norovirus GII (3 pairs) and *C. jejuni* (4 pairs).

**Supplementary Table S10.** The log_10_ minimum, mean and maximum values and standard deviations (SD) of fecal microbes before and after A) UV-LED disinfection, B) laboratory-scale biochar filtration and C) full-scale biochar filtration at the equine college.

| 1. UV-LED disinfection | | | | | | | | | | | | | | | | | | | | | | | | | | | | | |
| --- | --- | --- | --- | --- | --- | --- | --- | --- | --- | --- | --- | --- | --- | --- | --- | --- | --- | --- | --- | --- | --- | --- | --- | --- | --- | --- | --- | --- | --- |
| Tertiary treatment | | *E. coli* (log_10_ mpn/1000 mL) | | | | | | Intestinal enterococci  (log_10_ cfu/1000 mL) | | | | | | | | | | *C. perfringens* (log_10_ cfu/1000 mL) | | | | | | Somatic coliphages  (log_10_ pfu/1000 mL) | | | | | |
|  |  | Min. | Mean | | Max. | | SD | Min. | | Mean | | | Max. | | | SD | | Min. | Mean | | Max. | | SD | Min. | Mean | | Max. | | SD |
| Before UV-LED | | 2.86 | 3.67 | | 4.46 | | 0.76 | 2.36 | | 2.68 | | | 2.99 | | | 0.26 | | 3.08 | 3.41 | | 3.78 | | 0.27 | 2.02 | 2.35 | | 2.64 | | 0.27 |
| After UV-LED 300 L/h (N = 6*) | | 0.60 | 1.85 | | 2.93 | | 1.02 | 0.85 | | 1.34 | | | 1.72 | | | 0.29 | | 2.89 | 3.22 | | 3.57 | | 0.30 | 0.00 | 0.68 | | 1.18 | | 0.44 |
| After UV-LED 600 L/h (N = 6) | | 1.89 | 2.90 | | 3.96 | | 1.00 | 1.79 | | 2.02 | | | 2.38 | | | 0.22 | | 3.00 | 3.34 | | 3.72 | | 0.30 | 0.00 | 0.79 | | 1.56 | | 0.70 |
| 1. Laboratory-scale biochar filtration | | | | | | | | | | | | | | | | | | | | | | | | | | | | | |
| Tertiary treatment | | *E. coli* (log_10_ mpn/1000 mL) | | | | | | | | | | Intestinal enterococci (log_10_ cfu/1000 mL) | | | | | | | | | | Somatic coliphages (log_10_ pfu/1000 mL) | | | | | | | |
|  |  | Min. | | Mean | | Max. | | | SD | | | Min. | | | Mean | | | Max. | | SD | | Min. | | Mean | | Max. | | SD | |
| 50% wood-based biochar (N = 3) | Before | 3.57 | | 3.95 | | 4.38 | | | 0.33 | | | 2.81 | | | 3.27 | | | 3.56 | | 0.33 | | NA | | NA | | NA | | NA | |
|  | After | 3.00 | | 3.31 | | 3.79 | | | 0.35 | | | 2.40 | | | 2.76 | | | 2.96 | | 0.26 | | NA | | NA | | NA | | NA | |
| 50% sludge-based biochar (N = 3) | Before | 3.57 | | 3.95 | | 4.38 | | | 0.33 | | | 2.81 | | | 3.27 | | | 3.56 | | 0.33 | | NA | | NA | | NA | | NA | |
|  | After | 2.99 | | 3.10 | | 3.30 | | | 0.15 | | | 2.28 | | | 2.47 | | | 2.61 | | 0.14 | | NA | | NA | | NA | | NA | |
| 70% wood-based biochar (N = 2) | Before | 2.73 | | 3.02 | | 3.30 | | | 0.28 | | | 1.89 | | | 2.13 | | | 2.38 | | 0.25 | | 2.41 | | 2.73 | | 3.05 | | 0.32 | |
|  | After | 2.49 | | 2.70 | | 2.91 | | | 0.21 | | | 1.72 | | | 1.90 | | | 2.08 | | 0.18 | | 2.32 | | 2.46 | | 2.59 | | 0.13 | |
| 30% wood-based biochar (N = 2) | Before | 2.73 | | 3.02 | | 3.30 | | | 0.28 | | | 1.89 | | | 2.13 | | | 2.38 | | 0.25 | | 2.41 | | 2.73 | | 3.05 | | 0.32 | |
|  | After | 2.43 | | 2.48 | | 2.53 | | | 0.05 | | | 1.65 | | | 1.72 | | | 1.78 | | 0.06 | | 2.28 | | 2.37 | | 2.46 | | 0.09 | |
| Sand filter (N = 5**) | Before | 2.73 | | 3.57 | | 4.38 | | | 0.55 | | | 1.89 | | | 2.81 | | | 3.56 | | 0.63 | | 2.41 | | 2.73 | | 3.05 | | 0.32 | |
|  | After | 2.36 | | 3.08 | | 3.79 | | | 0.56 | | | 1.78 | | | 2.46 | | | 3.15 | | 0.58 | | 2.15 | | 2.27 | | 2.40 | | 0.13 | |
| 1. Full-scale biochar filtration at the equine college | | | | | | | | | | | | | | | | | | | | | | | | | | | | | |
| Tertiary treatment | | *E. coli* (log_10_ mpn/1000 mL) | | | | | | Intestinal enterococci  (log_10_ cfu/1000 mL) | | | | | | | | | | *C. perfringens* (log_10_ cfu/1000 mL) | | | | | | Somatic coliphages  (log_10_ pfu/1000 mL) | | | | | |
|  |  | Min. | Mean | | Max. | | SD | Min. | | | Mean | | | Max. | | | SD | Min. | Mean | | Max. | | SD | Min. | Mean | | Max. | | SD |
| Before filtration | | 1.85 | 2.61 | | 3.69 | | 0.78 | 2.26 | | | 2.54 | | | 2.95 | | | 0.30 | 0.65 | 1.51 | | 2.18 | | 0.64 | 0.70 | 1.87 | | 3.61 | | 1.26 |
| After biochar filter (N = 3) | | 1.30 | 2.49 | | 3.74 | | 1.00 | 2.00 | | | 2.68 | | | 3.23 | | | 0.51 | 0.65 | 0.68 | | 0.70 | | 0.02 | 0.70 | 1.97 | | 1.16 | | 3.51 |
| After sand filter (N = 3) | | 1.85 | 2.66 | | 3.76 | | 0.81 | 2.49 | | | 2.99 | | | 3.40 | | | 0.38 | 0.65 | 1.09 | | 1.90 | | 0.58 | 0.70 | 1.94 | | 3.41 | | 1.12 |

N = Number of treated samples. *For intestinal enterococci, N = 5. **For somatic coliphages, N = 2. NA = Not analyzed

**Supplementary Table S11.** Mean values and standard deviations of phosphorus (P) and nitrogen (N) concentrations in wastewater samples run through the differentially composed pilot scale biochar filters.

| Tertiary treatment | Mean P (µg/L) before filtration | Mean P (µg/L) after filtration | Mean N (µg/L) before filtration | Mean N (µg/L) after filtration | n |
| --- | --- | --- | --- | --- | --- |
| 50% wood-based biochar filter | 120±20 | 50±20 | 25 000±7 000 | 23 000±9 400 | 3 |
| 50% sludge-based biochar filter | 120±20 | 410±240 | 25 000±7 000 | 16 000±4 200 | 3 |
| 70% wood-based biochar filter | 120±20 | 110±5 | 25 000±7 000 | 18 000±110 | 2 |
| 30% wood-based biochar filter | 120±20 | 61±7 | 25 000±7 000 | 20 000±4 000 | 2 |
| Sand filter | 120±20 | 39±11 | 25 000±7 000 | 25 000±10 000 | 5 |

n = number of samples tested.

**4. References**

Bustin, S. A., Benes, V., Garson, J. A., Hellemans, J., Huggett, J., Kubista, M., Mueller, R., Nolan, T., Pfaffl, M. W., Shipley, G. L., Vandesompele, J., & Wittwer, C. T. (2009). The MIQE Guidelines: Minimum Information for Publication of Quantitative Real-Time PCR Experiments. *Clinical Chemistry*, *55*(4), 611–622. https://doi.org/10.1373/clinchem.2008.112797

DeFlorio-Barker S., Arnold B.F., Sams E.A., Dufour A.P., Colford J.M., Weisberg S.B., Schiff K.C. & Wade T.J. 2018. Child environmental exposures to water and sand at the beach: Findings from studies of over 68,000 subjects at 12 beaches*. Journal of Exposure Science & Environmental Epidemiology 28*: 93–100. https://doi.org/10.1038/jes.2017.23

European Parliament and Council (EC). (2006). DIRECTIVE 2006/7/EC OF THE EUROPEAN PARLIAMENT AND OF THE COUNCIL of 15 February 2006 concerning the management of bathing water quality and repealing Directive 76/160/EEC. Directive 2006/7/EC.

Finnish Meteorological Institute. (2023). *Havaintojen lataus* [Computer software]. https://www.ilmatieteenlaitos.fi/havaintojen-lataus

Haugland, R. A., Varma, M., Sivaganesan, M., Kelty, C., Peed, L., & Shanks, O. C. (2010). Evaluation of genetic markers from the 16S rRNA gene V2 region for use in quantitative detection of selected Bacteroidales species and human fecal waste by qPCR. *Systematic and Applied Microbiology*, *33*(6), 348–357. https://doi.org/10.1016/j.syapm.2010.06.001

International Organization for Standardization (ISO) (2017). Microbiology of the food chain. Horizontal method for determination of hepatitis A virus and norovirus using real-time RT-PC. Part 1: Method for quantification (ISO 15216-1:2017). International Organization for Standardization, Technical Committee CEN/TC 463, *Microbiology of the food chain*.

Jothikumar, N., Cromeans, T. L., Hill, V. R., Lu, X., Sobsey, M. D., & Erdman, D. D. (2005). Quantitative real-time PCR assays for detection of human adenoviruses and identification of serotypes 40 and 41. *Applied and Environmental Microbiology*, *71*(6), 3131–3136. https://doi.org/10.1128/AEM.71.6.3131-3136.2005

Kauppinen, A., Martikainen, K., Matikka, V., Veijalainen, A.-M., Pitkänen, T., Heinonen-Tanski, H., & Miettinen, I. T. (2014). Sand filters for removal of microbes and nutrients from wastewater during a one-year pilot study in a cold temperate climate. *Journal of Environmental Management*, *133*, 206–213. https://doi.org/10.1016/j.jenvman.2013.12.008

Ministry of Social Affairs and Health Finland. Decree 354/2008. Sosiaali- ja terveysministeriön asetus pienten yleisten uimarantojen uimaveden laatuvaatimuksista ja valvonnasta. https://www.finlex.fi/fi/laki/alkup/2008/20080354

Oka, T., Katayama, K., Hansman, G. S., Kageyama, T., Ogawa, S., Wu, F.-T., White, P. A., & Takeda, N. (2006). Detection of human sapovirus by real-time reverse transcription-polymerase chain reaction. *Journal of Medical Virology*, *78*(10), 1347–1353. https://doi.org/10.1002/jmv.20699

Rytkönen, A., Tiwari, A., Hokajärvi, A.-M., Uusheimo, S., Vepsäläinen, A., Tulonen, T., & Pitkänen, T. (2021). The use of ribosomal RNA as a microbial source tracking target highlights the assay host-specificity requirement in water quality assessments. *Frontiers in Microbiology*, *12*, 673306. https://doi.org/10.3389/fmicb.2021.673306

Schill, W. B., & Mathes, M. V. (2008). Real-time PCR detection and quantification of nine potential sources of fecal contamination by analysis of mitochondrial cytochrome *b* targets. *Environmental Science & Technology*, *42*(14), 5229–5234. https://doi.org/10.1021/es800051z

Teunis, P. F. M., Moe, C. L., Liu, P., E. Miller, S., Lindesmith, L., Baric, R. S., Le Pendu, J., & Calderon, R. L. (2008). Norwalk virus: How infectious is it? *Journal of Medical Virology*, *80*(8), 1468–1476. https://doi.org/10.1002/jmv.21237

Teunis, P., Van Den Brandhof, W., Nauta, M., Wagenaar, J., Van Den Kerkhof, H., & Van Pelt, W. (2005). A reconsideration of the Campylobacter dose–response relation. Epidemiology and Infection, 133(4), 583–592. https://doi.org/10.1017/S0950268805003912

Uusheimo, S., Huotari, J., Tulonen, T., Aalto, S. L., Rissanen, A. J., & Arvola, L. (2018). High nitrogen removal in a constructed wetland receiving treated wastewater in a cold climate. *Environmental Science & Technology*, *52*(22), 13343–13350. https://doi.org/10.1021/acs.est.8b03032

World Health Organization (WHO). (2017). Guidelines for drinking-water quality: fourth edition incorporating the first addendum. World Health Organization. https://iris.who.int/handle/10665/254637
